# Transfer of tomato immune receptor Ve1 confers Ave1‐dependent *Verticillium* resistance in tobacco and cotton

**DOI:** 10.1111/pbi.12804

**Published:** 2017-11-15

**Authors:** Yin Song, Linlin Liu, Yidong Wang, Dirk‐Jan Valkenburg, Xianlong Zhang, Longfu Zhu, Bart P. H. J. Thomma

**Affiliations:** ^1^ Laboratory of Phytopathology Wageningen University Wageningen The Netherlands; ^2^ National Key Laboratory of Crop Genetic Improvement Huazhong Agricultural University Wuhan China

**Keywords:** receptor‐like protein, verticillium dahliae, vascular wilt, cell surface receptor, pathogen resistance

## Abstract

Verticillium wilts caused by soilborne fungal species of the *Verticillium* genus are economically important plant diseases that affect a wide range of host plants and are notoriously difficult to combat. Perception of pathogen(‐induced) ligands by plant immune receptors is a key component of plant innate immunity. In tomato, race‐specific resistance to Verticillium wilt is governed by the cell surface‐localized immune receptor Ve1 through recognition of the effector protein Ave1 that is secreted by race 1 strains of *Verticillium* spp. It was previously demonstrated that transgenic expression of tomato *Ve1* in the model plant *Arabidopsis thaliana* leads to Verticillium wilt resistance. Here, we investigated whether tomato *Ve1* can confer *Verticillium* resistance when expressed in the crop species tobacco (*Nicotiana tabcum*) and cotton (*Gossypium hirsutum*). We show that transgenic tobacco and cotton plants constitutively expressing tomato *Ve1* exhibit enhanced resistance against Verticillium wilt in an Ave1‐dependent manner. Thus, we demonstrate that the functionality of tomato *Ve1* in Verticillium wilt resistance through recognition of the *Verticillium* effector Ave1 is retained after transfer to tobacco and cotton, implying that the Ve1‐mediated immune signalling pathway is evolutionary conserved across these plant species. Moreover, our results suggest that transfer of tomato *Ve1* across sexually incompatible plant species can be exploited in breeding programmes to engineer Verticillium wilt resistance.

## Introduction

In order to activate immune responses to ward off invading microorganisms, plants deploy immune receptors that detect pathogen invasion through sensing pathogen(‐induced) ligands (Cook *et al*., 2015; Dodds and Rathjen, 2010; Thomma *et al*., 2011). The recognition of such ligands results in the activation of defence responses, which are sometimes accompanied by a hypersensitive response (HR) in which plant tissue surrounding the site of attempted penetration is sacrificed to restrict further pathogen invasion. Based on structure and subcellular location, immune receptors fall into two major classes: cell surface‐localized receptors that comprise receptor kinases (RKs) and receptor‐like proteins (RLPs) that monitor the extracellular space, and cytoplasm‐localized nucleotide‐binding domain leucine‐rich repeat receptors (NLRs) that survey the intracellular environment (Rodriguez‐Moreno *et al*., 2017).

Verticillium wilts are vascular wilt diseases caused by soilborne fungal pathogens that belong to the *Verticillium* genus (Fradin and Thomma, 2006; Klimes *et al*., 2015). Although Verticillium wilt symptoms may vary considerably between plant hosts, the most frequently observed symptoms of Verticillium wilt include stunting, wilting, chlorosis, necrosis, vascular discoloration and early senescence (Fradin and Thomma, 2006). Within the *Verticillium* genus, *V. dahliae* is the most notorious pathogenic species that can infect hundreds of dicotyledonous hosts, including ecologically important plants and many high‐value crops worldwide (Fradin and Thomma, 2006; Klosterman *et al*., 2009). *V. albo‐atrum*,* V. alfalfae*,* V. nonalfalfae* and *V. longisporum* are also economically important vascular pathogens, albeit with narrower host ranges (Agrios, 2005; Depotter *et al*., 2016; Fradin and Thomma, 2006; Inderbitzin *et al*., 2011; Klosterman *et al*., 2009; Pegg and Brady, 2002).

Tobacco (*Nicotiana tabacum*) is an agriculturally and economically important Solanaceae crop that is grown in many regions worldwide. Verticillium wilt has been considered as a limiting factor for tobacco production globally, and is a serious disease of tobacco in New Zealand, Chile and Canada (Pegg and Brady, 2002), although the impact of Verticillium wilt on tobacco leaf yield and quality can be slightly reduced by careful farm practices. Verticillium wilt significantly impacts production of the cash crop cotton (*Gossypium* spp.) in cotton‐growing areas worldwide (Cai *et al*., 2009). To date, only few sea‐island cotton (*Gossypium barbadense*) cultivars have been shown to have some degree of resistance against Verticillium wilt (Cai *et al*., 2009), but sea‐island cotton is only cultivated on a small scale with <2% of cotton production globally (Chen *et al*., 2007). The most common cultivated cotton species, *Gossypium hirsutum* (upland cotton), is used for more than 95% of the annual cotton production worldwide (Chen *et al*., 2007). However, upland cotton cultivars with stably inherited resistance to Verticillium wilt are not available, and complex genetics of resistance to Verticillium wilt in upland cotton cultivars has limited the cross‐breeding of upland cotton cultivars with Verticillium wilt‐resistant sea‐island cotton germplasm (Cai *et al*., 2009). Therefore, Verticillium wilt‐resistant upland cotton cultivars are lacking (Cai *et al*., 2009).

Verticillium wilt diseases are difficult to control due to the long viability of the resting structures, the wide host range of the pathogens and the inability of fungicides to affect the pathogen once in the plant vascular system. The most sustainable manner to control Verticillium wilt diseases is the use of resistant cultivars (Fradin and Thomma, 2006; Klosterman *et al*., 2009). In tomato (*Solanum lycopersicum*), a single dominant locus that confers *Verticillium* resistance has been identified as the *Ve* locus, which controls *Verticillium* isolates that are assigned to race 1 whereas race 2 strains of *Verticillium* escape recognition (Pegg, 1974; Schaible *et al*., 1951). The *Ve* locus contains two closely linked and inversely oriented genes, *Ve1* and *Ve2*, both of which encode extracellular leucine‐rich repeat (eLRR) RLPs (Kawchuk *et al*., 2001; Wang *et al*., 2010). Of these, only *Ve1* was found to confer resistance against race 1 isolates of *Verticillium* in tomato (Fradin *et al*., 2009). Interestingly, interfamily transfer of *Ve1* from tomato to *Arabidopsis thaliana* leads to race‐specific *Verticillium* resistance in the latter species (Fradin *et al*., 2011, 2014; Zhang *et al*., 2014), implying that the underlying immune signalling cascade is evolutionary conserved (Fradin *et al*., 2011; Thomma *et al*., 2011). Moreover, homologs of tomato Ve1 that have the potential to recognize race 1 strains of *V. dahliae* have been characterized in other diverse plant species, suggesting an ancient origin of the tomato immune receptor Ve1 (Song *et al*., 2017a).

Through comparative population genomics of race 1 and race 2 strains of *V. dahliae*, the effector protein that activates Ve1‐mediated immunity was identified as Ave1 (for Avirulence on Ve1 tomato) (de Jonge *et al*., 2012). No allelic variation was found among the identified *Ave1* alleles from *V. dahliae* as well as from *V. alfalfae* and *V. nonalfalfae* (de Jonge *et al*., 2012; Song *et al*., 2017b). Interestingly, homologues of Ave1 were found in the bacterial plant pathogen *Xanthomonas axonopodis* pv. *citri* (XacPNP) and in the plant‐pathogenic fungi *Colletotrichum higginsianum* (ChAve1), *Cercospora beticola* (CbAve1), *Fusarium oxysporum* f. sp. *lycopersici* (FoAve1), as well as in plants (de Jonge *et al*., 2012). A few of these homologues are differentially recognized by tomato Ve1 in *Nicotiana tabacum* (de Jonge *et al*., 2012; Song *et al*., 2017b). Although the intrinsic function of Ave1 remains unknown, it is clear that Ave1 contributes to fungal virulence on susceptible plant genotypes (de Jonge *et al*., 2012).

Plant immune receptors are pivotal elements of the plant immune system that act as sentinels against pathogens. Engineering plants via transfer of immune components, such as plant immune receptors, have the potential to improve disease resistance in crops (Rodriguez‐Moreno *et al*., 2017). Previous reports showed that the transfer of individual cell surface immune receptors into crops confers enhanced disease resistance against diverse pathogens, including bacteria, fungi and oomycetes. For example, transfer of the *Arabidopsis* cell surface immune receptor EFR results in responsiveness to bacterial elongation factor Tu and bacterial resistance in tomato (Lacombe *et al*., 2010), rice (Lu *et al*., 2015; Schwessinger *et al*., 2015) and wheat (Schoonbeek *et al*., 2015). Similarly, introduction of the rice Xa21 confers bacterial resistance in sweet orange (Mendes *et al*., 2010), tomato (Afroz *et al*., 2011) and banana (Tripathi *et al*., 2014). Heterologous expression of the *Nicotiana benthamiana FLS2* in citrus leads to increased disease resistance to citrus canker (Hao *et al*., 2016). Moreover, the *Arabidopsis* DORN1/LecRK‐I.9 enhances resistance to *Phytophthora infestans* in potato (Bouwmeester *et al*., 2014). Finally, ectopic expression of the *Arabidopsis RLP23* in potato plants enhances immunity to the oomycete and fungal plant pathogens *P. infestans* and *Sclerotinia sclerotiorum* (Albert *et al*., 2015). In this study, we investigated whether the immune receptor gene *Ve1* can confer Verticillium wilt resistance when transferred from tomato to the closely related crop species tobacco (*N. tabacum* cv. Samsun) and the distantly related crop species cotton (*G. hirsutum*).

## Results

### Generation of *Ve1*‐expressing *Nicotiana tabacum* plants

**Figure 1:**
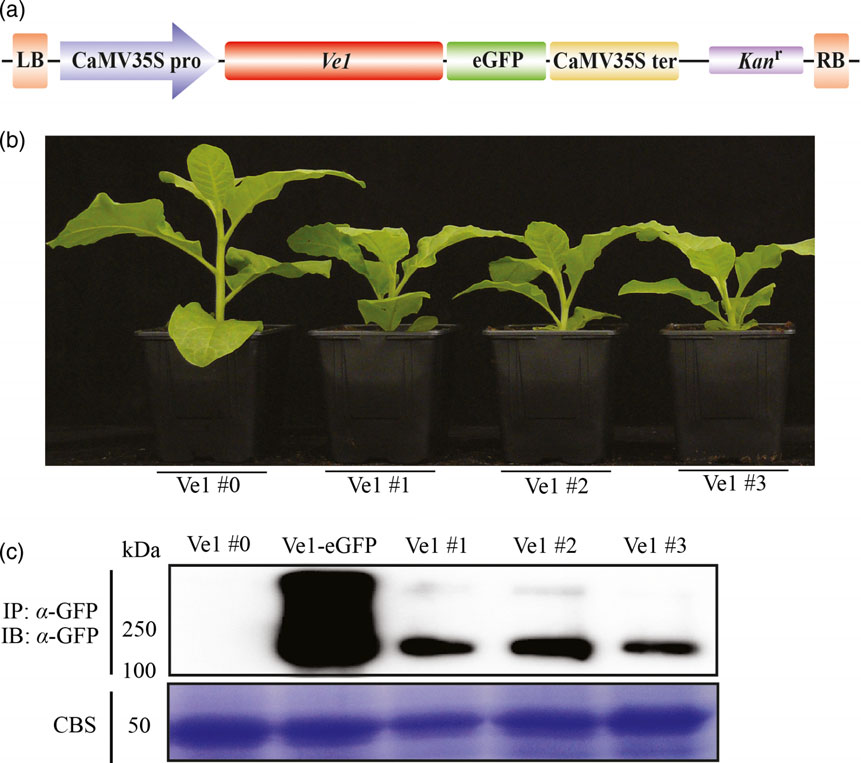
Generation and characterization of *Ve1*‐transgenic *Nicotiana tabacum* lines. (a) Schematic representation of the T‐DNA region of the binary vector pSol2095_Ve1 used for tobacco transformation. CaMV35Spro: CaMV35S promoter, eGFP: enhanced green fluorescent protein, CaMV35Ster: CaMV35S terminator; *Kan* ^*r*^: kanamycin resistance gene, LB and RB: left and right T‐DNA borders, respectively. (b) Typical appearance of 6‐week‐old plants of four independent tobacco lines (Ve1 #0, #1, #2 and #3). (c) Accumulation of eGFP‐tagged Ve1 protein (~144 kDa) in leaves of the four independent *Ve1* transgenic tobacco lines and wild‐type tobacco cv. Samsun transiently expressing the eGFP‐tagged Ve1 fusion protein (Ve1‐eGFP). Total protein extracts of transformed leaf tissue were subjected to immunoprecipitation (IP) using α‐GFP affinity beads. Proteins were subjected to sodium dodecyl sulphate–polyacrylamide electrophoresis (SDS/PAGE) and immunoblotted (IB) using α‐GFP antibody. Coomassie blue staining (CBS) of the blot containing total protein extracts is shown as a loading control based on the 50‐kDa RuBisCo (ribulose‐1,5‐bisphosphate carboxylase/oxygenase) band.

Previously, it was shown that co‐expression of *Ve1* and *Ave1* by agroinfiltration induces an HR in *N. tabacum* (Zhang *et al*., 2013a), suggesting that required signalling components acting downstream of tomato Ve1 are functionally conserved in tobacco. To further test whether tomato Ve1 can confer resistance to race 1 *Verticillium* spp., transgenic tobacco lines expressing tomato *Ve1* were generated. The binary plasmid pSol2095_Ve1 encoding C‐terminally eGFP‐tagged Ve1 (Zhang *et al*., 2013a; Figure 1a) was transferred to *N. tabacum* cv. Samsun via *Agrobacterium*‐mediated transformation. Primary transformants were selected in tissue culture by their ability to regenerate in the presence of kanamycin, and eight independent T0 transformation events expressing *Ve1* were obtained (Figure S1a).

Intriguingly, 45 of 56 progeny (T1 plants) derived from the eight T0 transformation events were significantly smaller in size when compared to the parental line, while 11 plants displayed a normal stature (Figure S1b,c). We assumed these 45 are *Ve1*‐transgenic plants while the 11 correspond to segregating wild‐type plants. To assay whether Ve1 protein accumulated in these plants, we isolated proteins from a line (Ve1 #0) that exhibited normal growth and three lines (Ve1 #1, #2 and #3) that showed a dwarfed phenotype (Figure 1b) and performed immunoblotting analysis using anti‐GFP‐HRP antibody. This analysis showed that eGFP‐tagged Ve1 protein was clearly detected in the three dwarfed lines, but not in the line that displays a normal stature (Figure 1c), suggesting that dwarfing of these tobacco lines is due to Ve1 expression. Nevertheless, the progeny of these three dwarfed lines was used for further assays to assess the contribution of Ve1 expression to Verticillium wilt resistance.

### Generation of isogenic *Verticillium* genotypes lacking or expressing *Ave1*

To identify *Verticillium* strains that can be used for inoculation assays on tobacco, six strains, comprising *V. dahliae* strains JR2 and St14.01, *V. nonalfalfae* strains Vna5431, CBS385.91 and Vna1, and *V. alfalfae* strain Va2 (Table S1) were inoculated onto wild‐type tobacco cv. Samsun plants, and Verticillium wilt symptoms were scored up to 14 days postinoculation (dpi). As anticipated, the various *Verticillium* strains caused different degrees of Verticillium wilt symptoms on these plants (Figure S2a). Among these six *Verticillium* genotypes, *V. alfalfae* strain Va2 and *V. nonalfalfae* strain Vna5431 caused the most severe disease symptoms (Figure S2a,b) and were selected for Verticillium wilt disease assays on tobacco. As *V. nonalfalfae* Vna5431 carries the *Ave1* gene, it belongs to race 1, while *V. alfalfae* Va2 belongs to race 2 as it lacks *Ave1* (Figure S2c).

To thoroughly investigate Ve1‐mediated Verticillium wilt resistance in tobacco, isogenic *Verticillium* genotypes lacking or expressing *Ave1* are required. To this end, we deleted *Ave1* from the genome of *V. nonalfalfae* Vna5431 and simultaneously introduced *Ave1* into *V. alfalfae* Va2 genome. Subsequently, the wild‐type *V. nonalfalfae* strain Vna5431 and two independent *Ave1* deletion mutants were used to inoculate *Ve1* tomato plants and tomato plants that lack *Ve1* (Figure S3a). As expected, targeted deletion of *Ave1* resulted in gain of virulence on *Ve1* tomato plants (Figure S3b,c). Moreover, these *Ave1* deletion mutants displayed reduced virulence on tomato plants lacking *Ve1* when compared to the corresponding wild‐type *V. nonalfalfae* strain Vna5431 (Figure S3b,d). These results show that, also for *V. nonalfalfae* strain, Vna5431 Ave1 acts as a virulence factor on tomato, and confirm that deletion of *Ave1* leads to escape of Ve1‐mediated resistance.

Simultaneously, the wild‐type *V. alfalfae* strain Va2 and two independent *Ave1* expression strains were inoculated onto tomato plants that express or lack *Ve1* (Figure S4a). However, the wild‐type *V. alfalfae* strain Va2, as well as the two *Ave1*‐expressing *V. alfalfae* strains, failed to cause visible disease symptoms on tomato plants (Figure S4b,d,e), suggesting that the *V. alfalfa*e strain Va2 does not have the capacity to infect tomato. Subsequently, we inoculated these strains on *N. glutinosa*, which is resistant to race 1 strains of *Verticillium* due to the occurrence of an endogenous *Ve1* allele (Song *et al*., 2017a; Zhang *et al*., 2013a). As expected, *V. alfalfae* strain Va2 was able to infect *N. glutinosa* plants, while the *Ave1* expression strains failed to cause infection (Figure S4c,f). These results reveal that ectopic expression of *Ave1* in *V. alfalfae* strain Va2 can activate Ve1‐mediated resistance against Verticillium wilt.

### Ave1 acts as a virulence factor on tobacco

It was previously determined that Ave1 acts as a virulence factor of *V. dahliae* on tomato and *A. thaliana* (de Jonge *et al*., 2012). To investigate the contribution of *Ave1* to *Verticillium* virulence on tobacco, isogenic *Ave1* mutants and the corresponding wild‐type *Verticillium* strain were inoculated onto tobacco cv. Samsun plants. Interestingly, *Ave1* deletion strains of *V. nonalfalfae* Vna5431 displayed significantly reduced virulence on tobacco plants when compared with the corresponding wild‐type *V. nonalfalfae* strain Vna5431 at 21 dpi (Figure S5a), as inoculation with the *Ave1* deletion strains resulted in reduced stunting (Figure S5b) and compromised fungal colonization (Figure S5c). Conversely, the two *Ave1* expression strains in *V. alfalfae* Va2 showed clearly increased aggressiveness on tobacco plants when compared with the corresponding wild‐type strain (Figure S5d–f). These experiments show that Ave1 acts as a virulence factor on tobacco plants.

### Tomato Ve1 confers Ave1‐dependent Verticillium wilt resistance in tobacco

**Figure 2:**
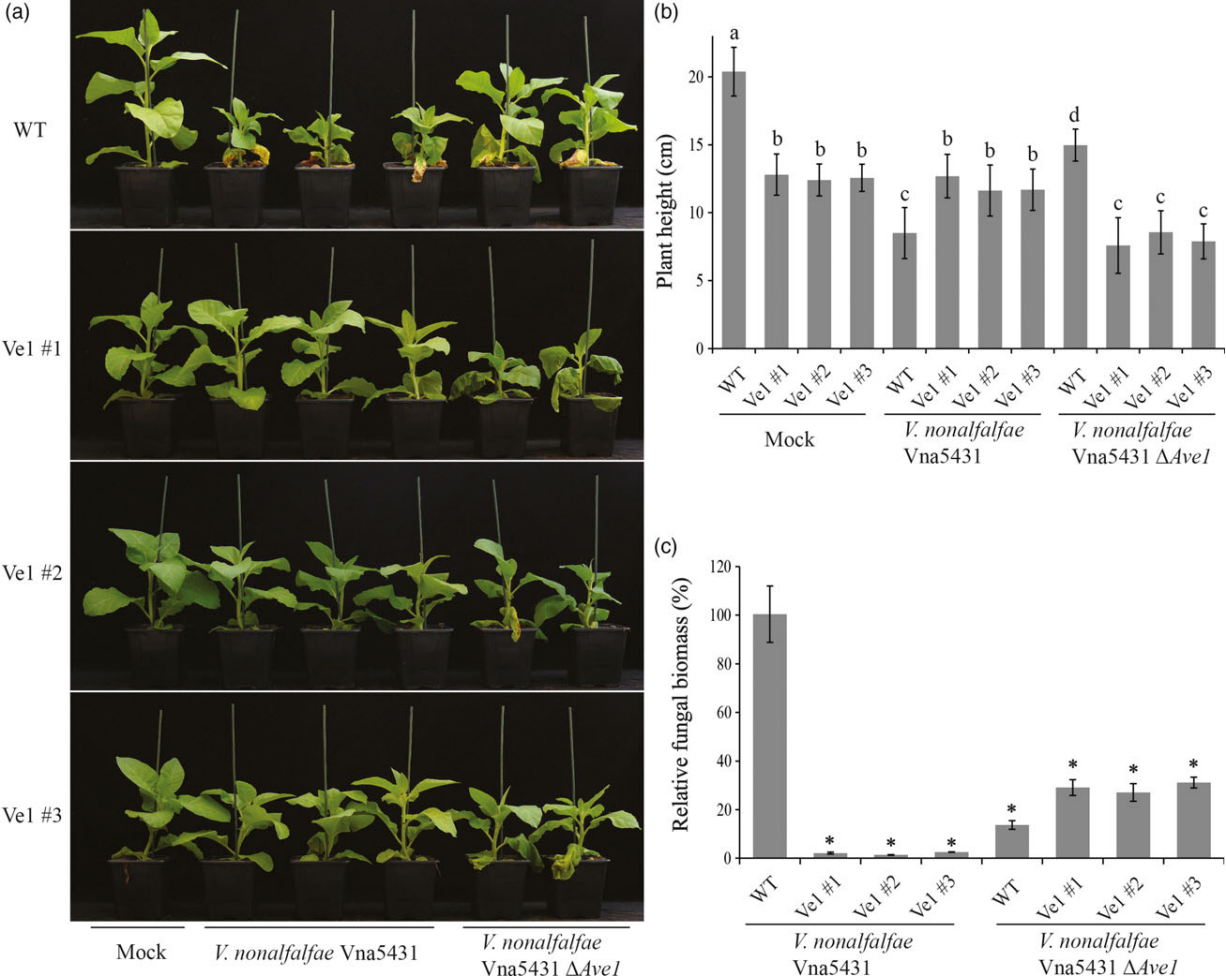
Tobacco plants expressing *Ve1* show Ave1‐triggered resistance against *Verticillium nonalfalfae*. (a) Typical appearance of wild‐type tobacco cultivar Samsun plants (WT) and three independent *Ve1* transgenic tobacco plants (#1, #2 and #3) that were engineered to express tomato *Ve1* upon mock inoculation, inoculation with *Ave1*‐carrying *V. nonalfalfae* Vna5431 or an *Ave1* deletion strain of *V. nonalfalfae* Vna5431 (Δ*Ave1*) at 21 days postinoculation (dpi). Inoculation experiments were performed with at least 16 plants for each fungal strain and independently repeated three times. (b) Quantification of *Verticillium*‐induced plant stunting at 21 dpi. Bars represent averages with standard deviation. Different letter labels indicate statistically significant differences (Student's *t*‐test; *P *<* *0.05). (c) Fungal biomass as determined with real‐time PCR at 21 dpi. Bars represent *Verticillium ITS* levels relative to tobacco *actin* levels (for equilibration) with standard deviation in a sample of three pooled plants. The fungal biomass in tobacco cv. Samsun plants upon inoculation with the wild‐type *V. nonalfalfae* strain Vna5431 is set to 100%. Asterisks indicate statistically significant differences when compared with tobacco cv. Samsun plants upon inoculation with the wild‐type *V. nonalfalfae* strain Vna5431 (Student's *t*‐test; *P *<* *0.05). The data shown are representative of three independent experiments.

**Figure 3:**
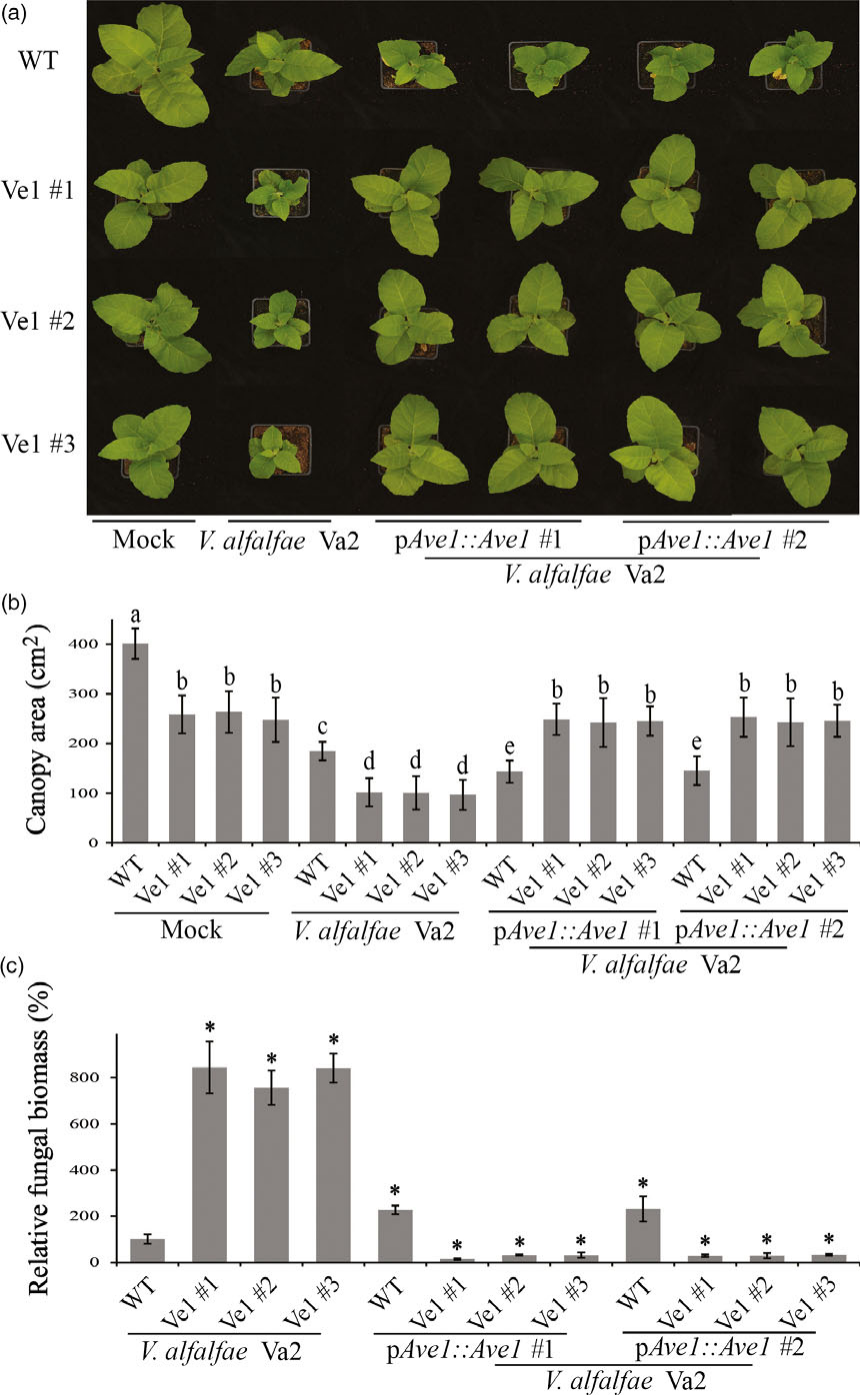
Tobacco plants expressing *Ve1* exhibit Ave1‐triggered resistance against *Verticillium alfalfae*. (a) Typical appearance of wild‐type tobacco cultivar Samsun plants (WT) and three independent *Ve1* transgenic tobacco plants (#1, #2 and #3) that were engineered to express tomato *Ve1* upon mock inoculation, inoculation with *V. alfalfae* Va2 or two *Ave1*‐expressing strains of *V. alfalfae* Va2 (p*A* *ve1::Ave1* #1 and #2) at 14 dpi. Inoculation experiments were performed with at least 16 plants for each fungal strain and independently repeated three times. (b) Quantification of the canopy area of tobacco plants at 14 dpi. Bars represent averages with standard deviation. Different letter labels indicate statistically significant differences (Student's *t*‐test; *P *<* *0.05). (c) Fungal biomass as determined with real‐time PCR at 14 dpi. Bars represent *Verticillium ITS* levels relative to tobacco *actin* levels (for equilibration) with standard deviation in a sample of three pooled plants. The fungal biomass in tobacco cv. Samsun plants upon inoculation with the wild‐type *V. alfalfae* strain Va2 is set to 100%. Asterisks indicate statistically significant differences when compared with tobacco cv. Samsun plants upon inoculation with the wild‐type *V. alfalfae* strain Va2 (Student's *t*‐test; *P *<* *0.05). The data shown are representative of three independent experiments.

To test whether constitutive *Ve1* expression in tobacco confers resistance against Verticillium wilt in an Ave1‐dependent manner, three independent *Ve1*‐transgenic lines (Ve1 #1, #2 and #3) as well as nontransgenic control plants were challenged with either the wild‐type race 1 *V. nonalfalfae* strain Vna5431, or an *Ave1* deletion mutant (*V. nonalfalfae* Vna5431Δ*Ave1*) and inspected for Verticillium wilt symptoms up to 21 dpi. Interestingly, *Ve1*‐transgenic tobacco plants were clearly more resistant to the race 1 *V. nonalfalfae* strain Vna5431, as significantly fewer Verticillium wilt symptoms developed when compared with nontransgenic controls (Figure 2a,b). Importantly, despite the fact that the *Ave1* deletion mutant of *V. nonalfalfae* Vna5431 displays compromised virulence on wild‐type tobacco plants, *Ve1*‐transgenic tobacco plants were clearly susceptible to this *Ave1* deletion mutant (Figure 2a,b). The phenotypes correlated with the degree of fungal colonization as determined by real‐time PCR (Figure 2c). Additionally, the three independent *Ve1*‐transgenic tobacco lines and nontransgenic controls were inoculated with either the wild‐type *V. alfalfae* strain Va2, or the two independent *Ave1* expression strains, and monitored for the development of Verticillium wilt symptoms at 14 dpi. Intriguingly, upon inoculation with the *Ave1* expression strains, no symptoms of Verticillium wilt were observed on the *Ve1*‐expressing tobacco plants, whereas the nontransgenic controls displayed clear symptoms of Verticillium wilt (Figure 3). Importantly, all *Ve1*‐transgenic lines were susceptible to the wild‐type *V. alfalfae* strain Va2. Collectively, these data show that tobacco plants expressing *Ve1* display enhanced Verticillium wilt resistance in an Ave1‐dependent manner.

### Generation of isogenic *V. dahliae* strains lacking *Ave1*

Verticillium wilt of cotton is mostly caused by *V. dahliae*, and thus far effective tools to control Verticillium wilt in cotton are lacking (Cai *et al*., 2009). In a previous attempt to investigate whether tomato *Ve1* can confer resistance against Verticillium wilt in cotton, transgenic cotton (*G. hirsutum* cv. YZ‐1) lines that express tomato *Ve1* were generated, but no increased Verticillium wilt resistance was observed (Liu *et al*., 2014). However, it was realized later on that the *V. dahliae* strains used in this study did not contain *Ave1* (Liu *et al*., 2014).

To re‐address the potential value of *Ve1* to engineer Verticillium wilt resistance in cotton, we pursued *Ave1*‐carrying *V. dahliae* that can cause clear Verticillium wilt symptoms on *G. hirsutum* cv. YZ‐1 plants. To this end, we tested *V. dahliae* strains JR2, V4, V991 and V117 (Table S1) on cotton cultivar YZ‐1, and the development of Verticillium wilt symptoms was monitored at 21 dpi. As expected, differential degrees of Verticillium wilt symptoms were observed on these cotton plants (Figure S6a). Whereas *V. dahliae* strain JR2 that carries *Ave1* only induced mild symptoms on cotton, *V. dahliae* strain V4 that similarly carries *Ave1*, and *V. dahliae* strains V991 and V117 that both lack *Ave1* induced considerably stronger Verticillium wilt symptoms (Figure S6). Thus, race 1 *V. dahliae* strain V4 was selected for further assays on cotton.

To obtain an isogenic line that lacks *Ave1*, targeted replacement of *Ave1* in *V. dahliae* strain V4 through homologous recombination was pursed (Figure S7a). To test whether the *Ave1* deletion strains of *V. dahliae* V4 indeed overcome recognition by Ve1, two independent *Ave1* deletion strains were inoculated onto tomato plants that express or lack *Ve1* (Figure S7a). As expected, *Ve1* tomato plants that were inoculated with two independent *Ave1* deletion strains of *V. dahliae* V4 showed a similar disease phenotype as *Ve1* tomato plants inoculated with the *V. dahliae* JR2Δ*Ave1* strain (Figure S7b,c; de Jonge *et al*., 2012), whereas *Ve1* tomato plants inoculated with wild‐type *V. dahliae* strains V4 and JR2 resembled mock‐inoculated *Ve1* tomato plants (Figure S7b,c). Moreover, the *Ave1* deletion strains of *V. dahliae* strain V4 displayed significantly reduced virulence on susceptible tomato plants when compared with the corresponding wild‐type race 1 *V. dahliae* strain V4 (Figure S7b,d). These results are in line with previous results that show that Ave1 acts as a virulence factor on tomato, and confirm that deletion of *Ave1* leads to escape of Ve1‐mediated Verticillium wilt resistance (de Jonge *et al*., 2012).

### Ave1 acts as a virulence factor on cotton

To investigate whether Ave1 acts as a virulence factor on cotton, two independent *Ave1* deletion strains and the corresponding wild‐type strain V4 were used to inoculate cotton cv. YZ‐1 plants. Interestingly, the *Ave1* deletion strains of *V. dahliae* V4 displayed clearly reduced virulence on wild‐type cotton plants when compared with the corresponding wild‐type strain up to 28 dpi (Figure S8a), as inoculation with *Ave1* deletion mutants resulted in significantly reduced stunting (Figure S8b) and compromised fungal colonization (Figure S8c). This assay demonstrates that Ave1 acts as a virulence factor also on cotton.

### Cotton plants expressing *Ve1* exhibit enhanced Verticillium wilt resistance in an Ave1‐dependent manner

**Figure 4:**
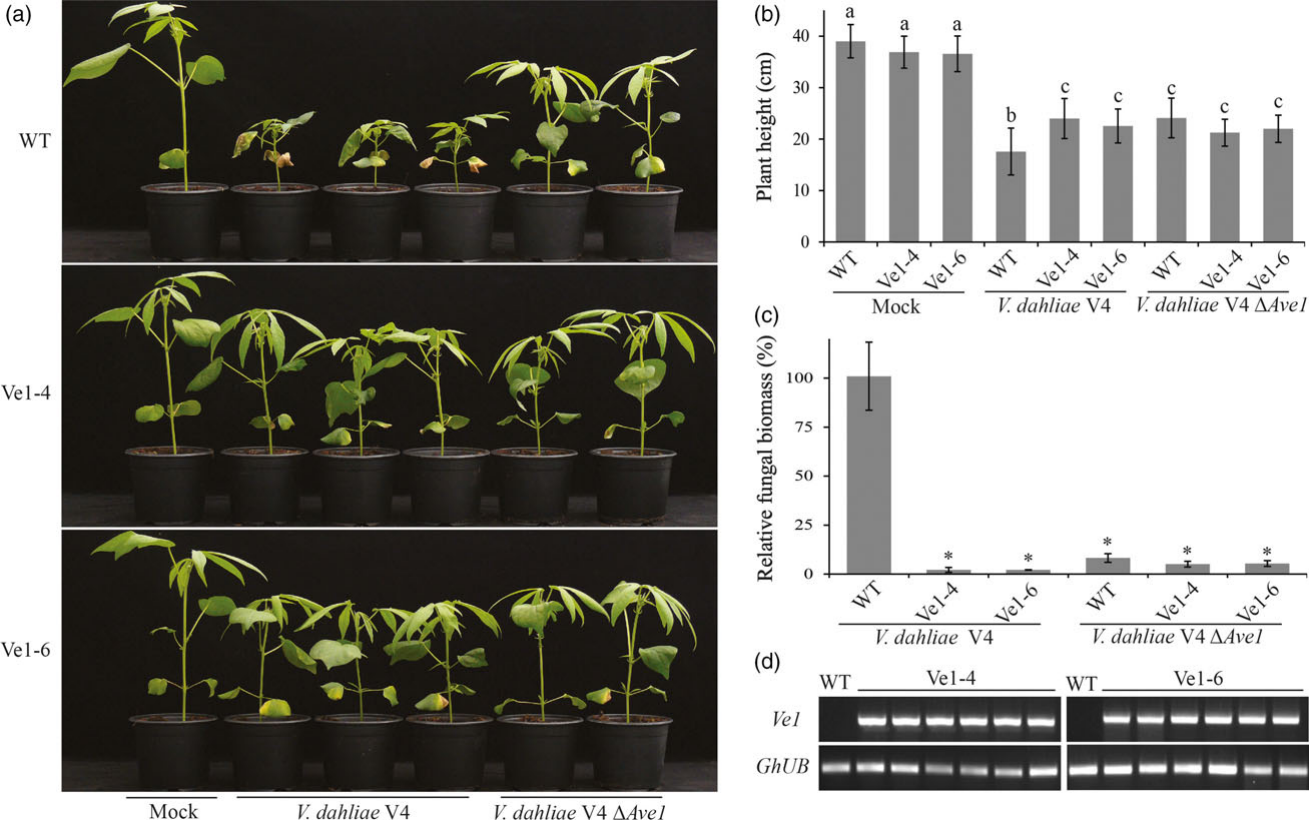
Cotton plants expressing *Ve1* display Ave1‐triggered resistance against *Verticillium dahliae*. (a) Typical appearance of wild‐type cotton cultivar YZ‐1 plants (WT) and two independent *Ve1* transgenic cotton plants (Ve1‐4 and Ve1‐6) upon mock inoculation, inoculation with *Ave1*‐carrying *V. dahliae* V4 or an *Ave1* deletion strain of *V. dahliae* V4 (Δ*Ave1*) at 28 dpi. Inoculation experiments were performed with at least 20 plants for each fungal strain and independently repeated three times. (b) Quantification of *Verticillium*‐induced plant stunting at 28 dpi. Bars represent averages with standard deviation. Different letter labels indicate statistically significant differences (Student's *t*‐test; *P *<* *0.05). (c) Fungal biomass as determined with real‐time PCR at 28 dpi. Bars represent *Verticillium ITS* levels relative to cotton *ubiquitin* levels (for equilibration) with standard deviation in a sample of three pooled plants. The fungal biomass in cotton cv. YZ‐1 plants upon inoculation with the wild‐type *V. dahliae* strain V4 is set to 100%. Asterisks indicate statistically significant differences when compared with cotton cv. YZ‐1 plants upon inoculation with the wild‐type *V. dahliae* strain V4 (Student's *t*‐test; *P *<* *0.05). (d) Expression of tomato *Ve1* in individual transgenic cotton plants and nontransgenic controls of cotton cv. YZ‐1 (WT) as detected with reverse transcription‐PCR (RT‐PCR). As an endogenous control, a fragment of the cotton *ubiquitin* gene (*GhUB*) was amplified. The data shown are representative of three independent experiments.

**Figure 5:**
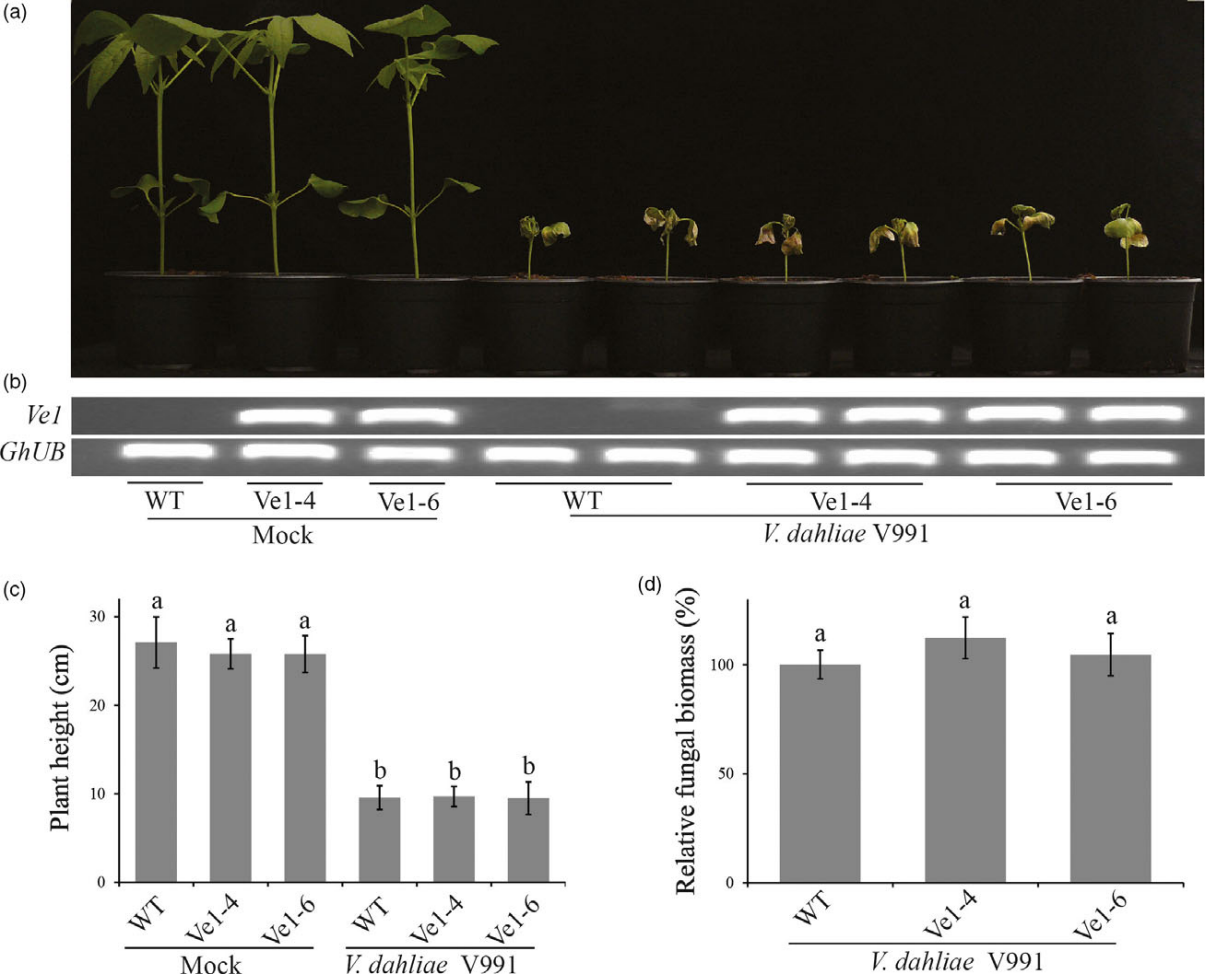
*Ve1*‐transgenic and nontransgenic cotton plants are equally susceptible to *Verticillium dahliae* lacking *Ave1*. (a) Typical appearance of wild‐type cotton cultivar YZ‐1 plants (WT) and transgenic cotton plants expressing tomato *Ve1* upon mock inoculation or inoculation with *V. dahliae* strain V991 at 28 dpi. Inoculation experiments were performed with at least 20 plants for *V. dahliae* strain V991 and independently repeated three times. (b) Expression of tomato *Ve1* in individual cotton plants from wild‐type controls and transgenic lines as detected with reverse transcription‐PCR (RT‐PCR). As an endogenous control, a fragment of the cotton *ubiquitin* gene (*GhUB*) was amplified. (c) Quantification of *Verticillium*‐induced plant stunting at 28 dpi. Bars represent averages with standard deviation. Different letter labels indicate statistically significant differences (Student's *t*‐test; *P *<* *0.05). (d) Fungal biomass as determined with real‐time PCR at 28 dpi. Bars represent *Verticillium ITS* levels relative to cotton *ubiquitin* levels (for equilibration) with standard deviation in a sample of three pooled plants. The fungal biomass in cotton cv. YZ‐1 plants upon inoculation with the *V. dahliae* strain V991 is set to 100%. Same letter labels indicate no statistically significant differences (Student's *t*‐test; *P *>* *0.05). The data shown are representative of three independent experiments.

To investigate whether cotton plants constitutively expressing tomato *Ve1* display enhanced resistance against race 1 *V. dahliae*, two *Ve1*‐trangenic lines (Ve1‐4 and Ve1‐6) as well as nontransgenic control plants were inoculated with either the race 1 *V. dahliae* strain V4 or an *Ave1* deletion mutant (*V. dahliae* V4Δ*Ave1*), and monitored for Verticillium wilt symptoms up to 28 dpi. As expected, clear Verticillium wilt symptoms were observed on nontransgenic plants upon inoculation with *V. dahliae* strain V4 and with the corresponding *Ave1* deletion mutant (Figure 4a–c), despite the observation that *Ave1* deletion compromises virulence on cotton. Interestingly, *Ve1*‐expressing cotton plants exhibited significantly enhanced resistance against *V. dahliae* strain V4, as less Verticillium wilt symptoms were observed when compared with nontransgenic controls (Figure 4). When the two *Ve1*‐transgenic lines and nontransgenic controls were challenged with *V. dahliae* strain V991 that does not carry *Ave1*,* Ve1*‐expressing cotton lines were as susceptible as nontransgenic controls (Figure 5), confirming that the enhanced Verticillium wilt resistance upon *Ve1* expression is Ave1‐dependent. Taken together, these data demonstrate that transfer of tomato immune receptor Ve1 into cotton confers Ave1‐dependent Verticillium wilt resistance.

## Discussion

Major management strategies for Verticillium wilt diseases in crops include chemical and biological control and cultivation practices, and for the use of disease‐resistant cultivars (Fradin and Thomma, 2006; Klosterman *et al*., 2009). Although chemical control has been proven to be successful for many diseases and pests, no truly effective fungicides are commercially available to control Verticillium wilt diseases once plants have been infected (Fradin and Thomma, 2006; Klosterman *et al*., 2009). Biocontrol measures and cultivation practices for controlling Verticillium wilts are time‐consuming and laborious, and control effectiveness largely depends on the field conditions. Therefore, breeding for disease‐resistant cultivars has been considered as the most sustainable approach to control Verticillium wilt diseases in crops (Fradin and Thomma, 2006; Klosterman *et al*., 2009). Presently, genetic resistance against *Verticillium* spp. has been described in several plant species, including tomato, potato, hop, alfalfa, cotton, strawberry, sunflower and lettuce (Antanaviciute *et al*., 2015; Barrow, 1970; Bolek *et al*., 2005; Christopoulou *et al*., 2015; Hayes *et al*., 2011; Jakse *et al*., 2013; Lynch *et al*., 1997; Mert *et al*., 2005; Putt, 1964; Schaible *et al*., 1951; Simko *et al*., 2004; Wang *et al*., 2008; Yang *et al*., 2008). However, only tomato *Ve1* has been cloned and characterized as a dominant gene responsible for race 1 Verticillium wilt resistance (Fradin *et al*., 2009; Kawchuk *et al*., 2001). We previously reported that homologues of tomato *Ve1* occur widespread in phylogenetically distant plant species (Song *et al*., 2017a). However, despite being widespread, *Ve1* homologues occur in a scattered fashion throughout plant phylogeny. For instance, a functional *Ve1* allele was identified in *N. glutinosa*, but not in other species within the genus *Nicotiana* (Song *et al*., 2017a; Zhang *et al*., 2013a). This finding implies that, most likely, many plant species lost their functional *Ve1* homologues, but underlying immune signalling cascade may have been retained. Indeed, we have previously shown that heterologous expression of tomato *Ve1* in the model plant *A. thaliana* that does not normally respond to Ave1 results in resistance against race 1 *Verticillium* spp. (Fradin *et al*., 2011; de Jonge *et al*., 2012; Zhang *et al*., 2013b). In this study, we investigated whether tomato *Ve1* can confer Verticillium wilt resistance when expressed in the crop species tobacco and cotton. We show that transgenic tobacco and cotton plants constitutively expressing tomato *Ve1* display enhanced resistance against Verticillium wilt in an Ave1‐dependent manner. Thus, our results reveal that the functionality of tomato *Ve1* in resistance against Verticillium wilt through recognition of the *Verticillium* effector Ave1 is retained after transfer to these plant species, and further support the view that the underlying immune signalling cascade mediated by Ve1 is retained in these plant species.

To date, several examples of transgenic expression of cell surface immune receptor genes resulting in enhanced disease resistance have been reported (Rodriguez‐Moreno *et al*., 2017). Although transgenic expression of such receptors enhanced disease resistance, in some cases it also has adverse effects on plant fitness, such as growth retardation or leaf necrosis (Bouwmeester *et al*., 2011, 2014; Wang *et al*., 2016). In this study, we observed that expression of tomato *Ve1* in tobacco caused stunted growth (Figures 1 and S1). A similar growth defect has previously been observed in *N. benthamiana* plants that constitutively express *Ve1* (Fradin, 2011). In contrast, potato (Kawchuk *et al*., 2001), tomato (Fradin *et al*., 2009), *A. thaliana* (Fradin *et al*., 2011) and cotton (Figures 4 and 5) plants expressing *Ve1* do not suffer from such growth defects. Based on these findings, we speculate that expression of tomato *Ve1* in the genus *Nicotiana* may lead to a constitutive activation of downstream signalling cascade of tomato Ve1 that causes growth retardation. Alternatively, a ligand that is endogenous to these tobacco genotypes is recognized, leading to immune signalling activation.

Previously, we have shown that Ave1 acts as a virulence factor on tomato as well as on *A. thaliana* (de Jonge *et al*., 2012). In the present study, we observed that targeted *Ave1* deletion results in significantly compromised virulence of *Verticillium* spp. on tobacco (Figure S5) and cotton (Figure S8), demonstrating that Ave1 acts as a virulence factor also on these plants. Previously, the bacterial homolog XacPNP from *X. axonopodis* pv. *citri* was characterized as a virulence factor on citrus trees (Gottig *et al*., 2008; Nembaware *et al*., 2004). More recently, Ave1 homologs from the fungal tomato wilt pathogen *F. oxysporum* f. sp. *lycopersici* (FoAve1), and the fungal sugar beet leaf spot pathogen *C. beticola* (CbAve1) were characterized as virulence factors too. Although the intrinsic function of the fungal Ave1 homologs remains enigmatic, XacPNP is thought to manipulate the physiology of the host through plant natriuretic peptide (PNP; immunological analogues of mammalian atrial natriuretic peptides) activity that affects water homoeostasis, stomatal opening and photosynthesis to promote bacterial proliferation (Garavaglia *et al*., 2010; Gottig *et al*., 2008). It is tempting to speculate that the fungal homologs promote virulence through a similar activity, but this remains to be demonstrated. In any case, the finding that Ave1 promotes virulence on tomato, tobacco, cotton as well as *A. thaliana* suggests that the molecular target of the effector is widely conserved in the plant kingdom.

In summary, our data demonstrate that transfer of tomato *Ve1* into the closely related crop species tobacco and the distantly related crop species leads to enhanced resistance against Verticillium wilt in an Ave1‐dependent manner. Given that Ave1 homologues were found in a number of pathogenic microbes (Gan *et al*., 2013; de Jonge *et al*., 2012; Nembaware *et al*., 2004), and these homologs were differentially recognized by tomato Ve1 (de Jonge *et al*., 2012; Song *et al*., 2017b), our findings may further broaden biotechnological avenues to exploit tomato Ve1 for engineering disease resistance in an Ave1(homolog)‐dependent manner, for instance through transfer or artificial evolution of tomato *Ve1*.

## Experimental procedures

### Plant materials and growth conditions

*Nicotiana tabacum* cv. Samsun, *N. glutinosa*, cotton (*Gossypium hirsutum* cv. YZ‐1) and tomato (*Solanum lycopersicum* cv. Moneymaker (*ve1*) or *35S::Ve1* tomato (*Ve1*); Fradin *et al*., 2009) plants were used in this study and grown in commercial potting soil (Horticoop, Bleiswijk, the Netherlands) under controlled greenhouse conditions (Unifarm, Wageningen, the Netherlands).

*Nicotiana tabacum* cv. Samsun seeds were surface‐sterilized by 70% ethanol and 1% commercial bleach, and grown on Murashige‐Skoog (MS) medium (4.4 g MS salt, 20 g sucrose and 8 g agar in 1 L) or MS medium supplemented with antibiotics in a conditioned growth chamber at 21°C/19°C during 16‐h/8‐h light/dark photoperiods, respectively, and a relative humidity of ~75%.

### Generation of *Ve1*‐transgenic plants

*Agrobacterium tumefaciens* GV3101 (pMP90) carrying the binary vector pSol2095_Ve1 to encode C‐terminally eGFP‐tagged Ve1 (Figure 1a; Zhang *et al*., 2013a) was used for transformation of tobacco *N. tabacum* cv. Samsun. Transformation was performed by the leaf disc method as previously described (Wang *et al*., 2016). The generated plantlets were transferred to half‐strength MS medium containing 200 mg/L kanamycin to allow root development. Upon root generation, plantlets were transferred into soil and grown in the greenhouse for seed production. Independent tobacco transformation lines were confirmed by PCR and reverse transcription‐PCR (RT‐PCR).

Two independent T0 generation cotton lines expressing tomato *Ve1* gene driven by the *cauliflower mosaic virus* 35S promoter (Ve1‐4 and Ve1‐6; Liu *et al*., 2014) were self‐pollinated to generate seeds. After two generations of selfing, T2 seeds were used for further experiments.

### Protein extraction, immunoprecipitation and immunoblotting

To test whether eGFP‐tagged Ve1 protein accumulated in transgenic tobacco lines, leaves of six‐week‐old transgenic tobacco lines were harvested and ground into a fine powder in liquid nitrogen. As a positive control, *A. tumefaciens* carrying the binary vector pSol2095_Ve1 was infiltrated into mature *N. tabacum* cv. Samsun leaves as described previously (Zhang *et al*., 2013a). Total proteins were extracted using extraction buffer (150 mm NaCl, 50 mm Tris‐HCl pH 8.0, 1.0% IGEPAL^®^ CA‐630 [NP‐40] (Sigma‐Aldrich Chemie BV, Zwijndrecht, the Netherlands) and one protease inhibitor cocktail tablet (Roche, Basel, Switzerland) per 50 mL extract buffer). Samples were centrifuged at 21 191 ***g*** for 20 min at 4°C, and then, 2 mL of supernatant was incubated with 10 μL (50% slurry) of GFP‐trap^®^_A beads (ChromoTek, Munich, Germany) at 4°C for 1 h. After incubation, GFP‐trap^®^_A beads with proteins were spun down by 110 ***g*** centrifugation and subsequently washed for six times in 1 mL extraction buffer. After each wash step, the GFP‐trap^®^_A beads were collected by 1000 rpm centrifugation. Proteins were released from GFP‐trap^®^_A beads by boiling for 5 min, separated on a 10% SDS‐PAGE gel and wet‐electroblotted onto PVDF membrane (Bio‐Rad, Hercules, CA). Accumulation of eGFP‐tagged Ve1 was detected by immunoblotting using anti‐GFP‐HRP antibody (Miltenyi Biotec, Bergisch Gladbach, Germany). SuperSignal^TM^ West Femto Maximum Sensitivity Substrate (Thermo Scientific, Waltham, MA) was used for signal development. Coomassie blue staining was used as loading control.

### Generation of *Ave1* mutant strains

*Verticillium* strains (Table S1) were grown on potato dextrose agar (PDA; Oxoid, Basingstoke, UK) at 22°C. The *Ave1* knockout construct pRF‐HU2_Ave1 that was described previously (de Jonge *et al*., 2012) was used to generate *Ave1* deletion mutants in *V. nonalfalfae* strain Vna 5431 and *V. dahliae* strain V4 (Table S1). The *Ave1* complementation construct pFBT 005_pAve1::Ave1 that was described earlier (Song *et al*., 2017b) was used to generate *Ave1* expression strains in *V. alfalfae* strain Va2 (Table S1).

*Agrobacterium tumefaciens*‐mediated *Verticillium* transformation was performed as described previously (Santhanam, 2012), and *Verticillium* deletion transformants were selected on PDA (Oxoid, Basingstoke, UK) containing 200 μg/mL cefotaxime and 50 μg/mL hygromycin (Duchefa, Haarlem, the Netherlands). *Ave1* expression transformants were selected on PDA supplemented with 200 μg/mL cefotaxime (Duchefa, Haarlem, the Netherlands) and 50 μg/mL nourseothricin sulphate (Sigma‐Aldrich Chemie BV, Zwijndrecht, the Netherlands). Putative *Verticillium* transformants were tested by PCR, and subsequent inoculation on *Ve1* tomato plants (*Ve1*) and tomato cultivar Moneymaker plants (*ve1*) (Fradin *et al*., 2009), or *N. glutionsa* plants carrying a functional *Ve1* homolog (Song *et al*., 2017a).

### Disease assays

*Verticillium* conidiospores were collected from 7‐ to 10‐day‐old cultures on PDA plates and washed with tap water. Disease assays were performed on tomato, tobacco and cotton plants using the root‐dipping inoculation method as previously described (Fradin *et al*., 2009). Briefly, 10‐day‐old *Ve1* or *ve1* tomato seedlings (for inoculation with *Verticillium Ave1* deletion strains), or four‐week‐old tobacco (*N. tabacum* cv. Samsun or *N. glutinosa*) plants or 10‐day‐old cotton seedlings were uprooted. Next, the roots were rinsed in water, dipped for 5 min in a suspension of 10^6^ conidiospores/mL water while the roots of mock plants (control) were dipped in tap water without conidiospores, and subsequently transplanted to fresh commercial potting soil (Horticoop, Bleiswijk, the Netherlands). Disease symptoms were scored up to 14 days postinoculation (dpi) (tomato, *N. glutinosa* and *N. tabacum* cv. Samsun), or 21 dpi (*N. tabacum* cv. Samsun), or 28 dpi (cotton). To this end, plants were photographed, and Image J was used to determine the canopy area (for quantification of stunting) while the rectilinear scale was used to measure the plant height (for quantification of growth). For fungal biomass quantification *in planta*, stems of three inoculated plants were harvested at 14 dpi (for *N. tabacum* cv. Samsun upon *V. alfalfae* inoculation), 21 dpi (for *N. tabacum* cv. Samsun upon *V. nonalfalfae* inoculation) or 28 dpi (cotton upon *V. dahliae* inoculation). The samples were ground into a fine powder in liquid nitrogen, and genomic DNA was isolated. Real‐time PCR was conducted using the fungus‐specific primers ITS‐F and ITS‐R (Table S2) with primers for tobacco *actin* (GenBank accession number: X69885; for *Verticillium*‐infected tobacco) or cotton *ubiquitin* (GenBank accession number: DQ116441; for *Verticillium*‐infected cotton) (Table S2) as an endogenous plant control, employing an ABI 7300 PCR system (Applied Biosystems, Foster City, CA) with the qPCR Core kit for SYBR Green I (Eurogentec Nederland BV, Maastricht, the Netherlands).

### Gene expression analysis

For the expression of *Ve1* in transgenic tobacco plants, leaves of six‐week‐old tobacco plants were harvested and ground into a fine powder in liquid nitrogen. Tobacco total RNA isolation and cDNA synthesis were performed as previously described (Song *et al*., 2017a). RT‐PCR was conducted using the primers Ve1‐F(PCR) and Ve1‐R(PCR) (Table S2), and *N. tabacum actin* gene (*NtACT*) (Table S2) was used as the endogenous control.

To check whether the *Ve1* gene is expressed in individual T2 cotton plants, leaves of five‐week‐old cotton plants were collected separately, flash frozen in liquid nitrogen and stored at −80°C for total RNA isolation. Cotton total RNA was isolated using Spectrum™ Plant Total RNA Kit (Sigma‐Aldrich Chemie BV, Zwijndrecht, the Netherlands) following the manufacturer's instructions. First‐strand cDNA synthesis was performed using M‐MLV reverse transcriptase system (Promega, Madison, WI). RT‐PCR was conducted with primers Ve1‐F(RT) and Ve1‐R(RT) (Table S2) in a total volume of 25 μL with 17.9 sterilized water, 5 μL 5× PCR buffer, 0.5 μL dNTPs, 0.5 μL of each primer, 0.1 μL GoTag DNA polymerase (Promega) and 1.0 μL of first‐strand cDNA. Primers GhUb‐F and GhUb‐R (Table S2) were used to amplify the cotton *ubiquitin* gene as endogenous loading control. PCR amplification consisted of an initial denaturation step of 95°C for 5 min, followed by denaturation at 95°C for 30 s, annealing at 55°C for 30 s and extension at 72°C for 40 s with 35 cycles. The resulting PCR products were subjected to agarose gel electrophoresis.

## Supporting information


**Figure S1** Characterisation of *Ve1*‐transgenic *Nicotiana tabacum* cv. Samsun plants.
**Figure S2** *Verticillium* strains induce differential degrees of Verticillium wilt symptoms on *N. tabacum* cv. Samsun plants.
**Figure S3** Analysis of *Ave1* deletion strains of *V. nonalfalfae* Vna5431.
**Figure S4** Analysis of ectopic expression *Ave1* strains in *V. alfalfae* Va2.
**Figure S5** Ave1 acts as a virulence factor on tobacco cv. Samsun plants.
**Figure S6** *V. dahliae* strains induce differential degrees of Verticillium wilt on cotton (*Gossypium hirsutum*) cv. YZ‐1 plants.
**Figure S7** Analysis of *Ave1* deletion strains of *V. dahliae* V4.
**Figure S8** Ave1 acts as a virulence factor on cotton plants.
**Table S1** *Verticillium* strains used in this study.
**Table S2** Primers used in this study.
