# Supplementary material for: Transfer of tomato immune receptor Ve1 confers Ave1‐dependent Verticillium resistance in tobacco and cotton
Source: Plant Biotechnol J. 2017 Nov 15;16(2):638–48. doi: 10.1111/pbi.12804 (PMC5787823; doi:10.1111/pbi.12804)
Supplement: Supplementary file 1 — Figure S1 Characterisation of Ve1‐transgenic Nicotiana tabacum cv. Samsun plants. Figure S2 Verticillium strains induce differential degrees of Verticillium wilt symptoms on N. tabacum cv. Samsun plants. Figure S3 Analysis of Ave1 deletion strains of V. nonalfalfae Vna5431. Figure S4 Analysis of ectopic expression Ave1 strains in V. alfalfae Va2. Figure S5 Ave1 acts as a virulence factor on tobacco cv. Samsun plants. Figure S6 V. dahliae strains induce differential degrees of Verticillium wilt on cotton (Gossypium hirsutum) cv. YZ‐1 plants. Figure S7 Analysis of Ave1 deletion strains of V. dahliae V4. Figure S8 Ave1 acts as a virulence factor on cotton plants. Table S1 Verticillium strains used in this study. Table S2 Primers used in this study. [file PBI-16-638-s001.docx]

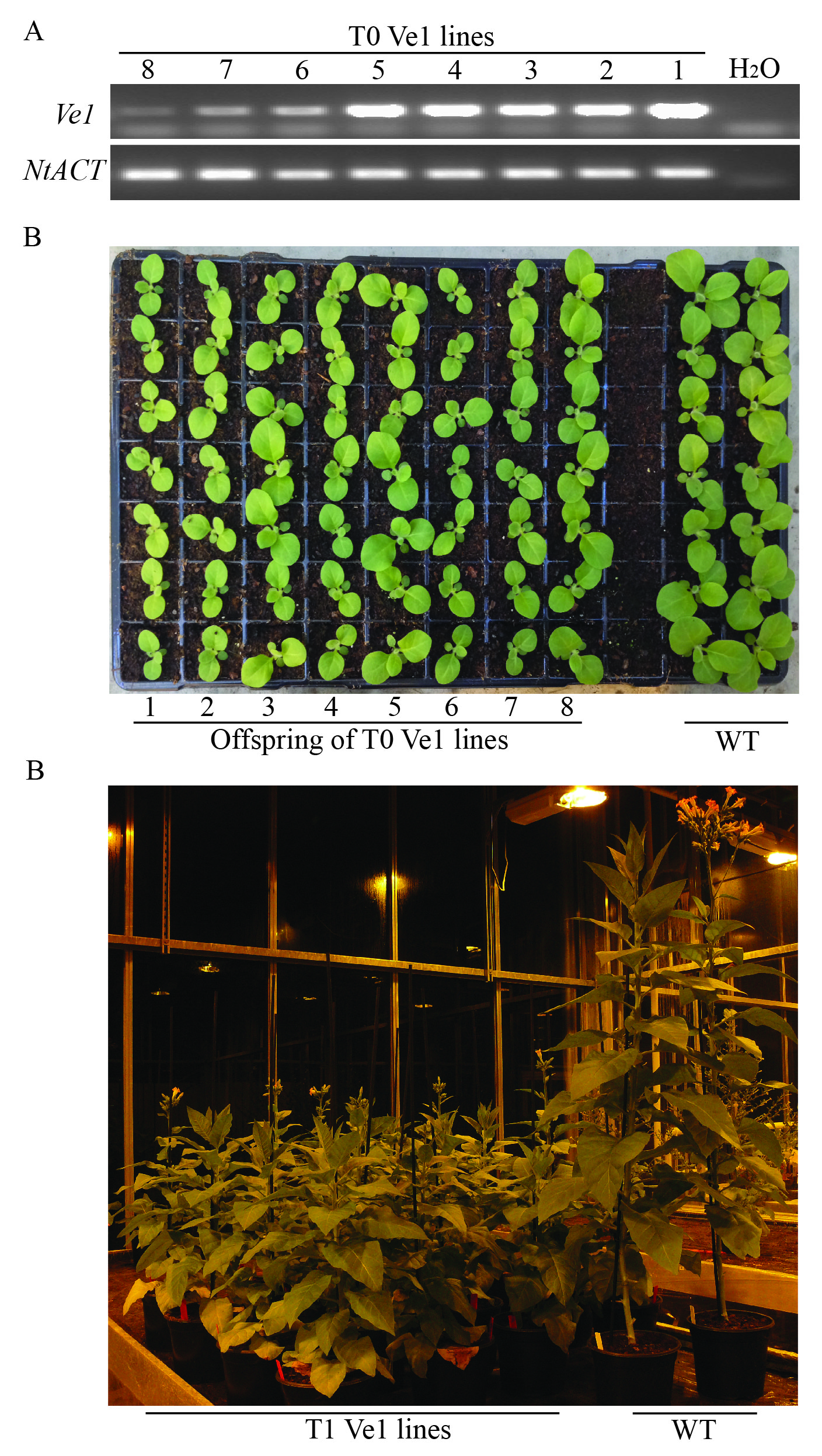


**Figure S1.** Characterisation of *Ve1*-transgenic *Nicotiana tabacum* cv. Samsun plants. (A) Transcripts of tomato *Ve1* in eight independent T0 transformation events were detected by reverse transcription-PCR (RT-PCR). As an endogenous control, a fragment of the *N. tabacum* *actin* gene (*NtACT*) was amplified. Water was used as a PCR control. (B) Morphology of four-week-old offspring from the eight T0 transformation events and the parental line. (C) Morphology of six-month-old T1 *Ve1*-transgenic tobacco lines and the recipient line tobacco cv. Samsun.


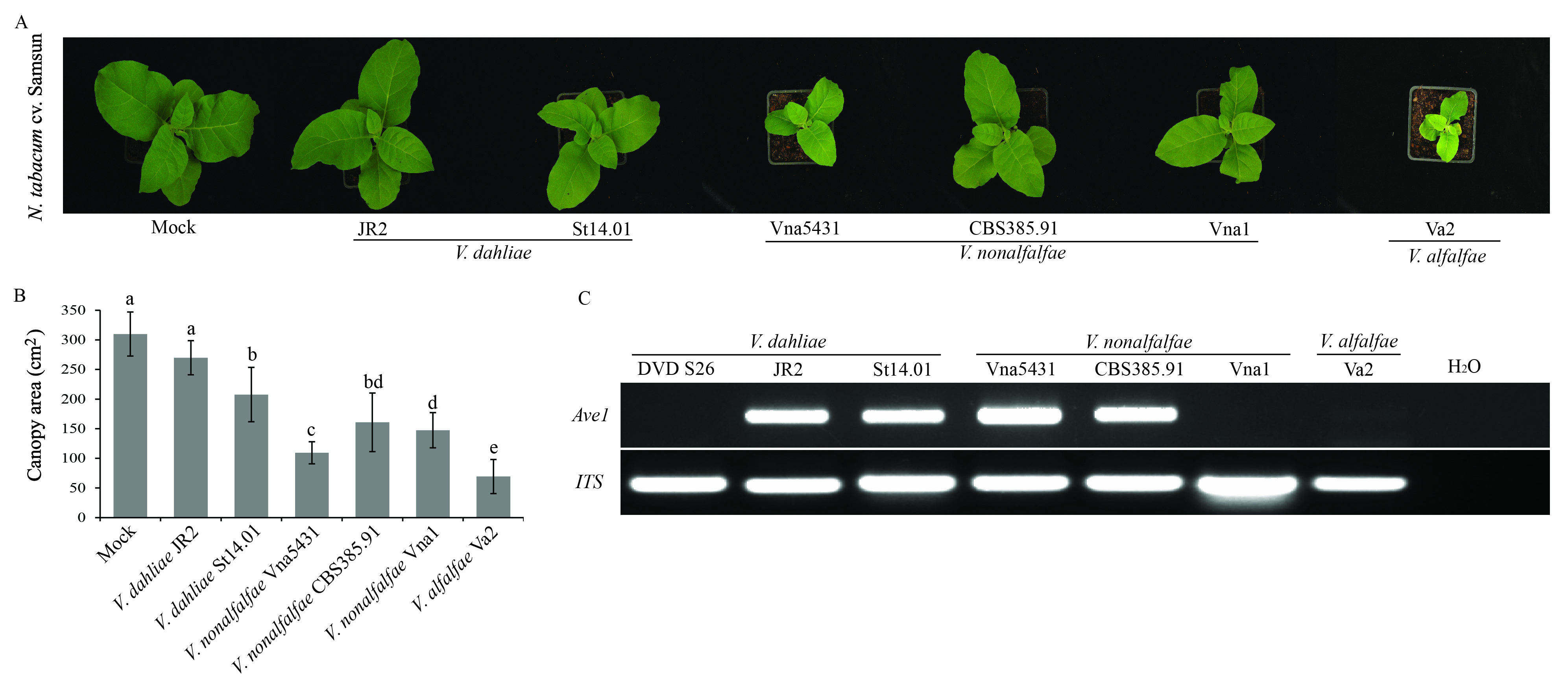


**Figure S2.** *Verticillium* strains induce differential degrees of Verticillium wilt symptoms on *N. tabacum* cv. Samsun plants. (A) Typical appearance of tobacco cv. Samsun plants upon mock-inoculation, inoculation with *V. dahliae* strains JR2 and St14.01, *V. nonalfalfae* strains Vna5431, CBS385.91 and Vna1, or *V. alfalfae* strain Va2 at 14 dpi. Inoculation experiments were performed with eight plants for each fungal strain and independently repeated twice. (B) Quantification of the canopy area of tobacco cv. Samsun plants at 14 dpi. Bars represent averages with standard deviation. Different letters indicate statistically significant differences (Student’s *t*-test; *P* < 0.05). (C) Presence of the full-length *Ave1* coding DNA sequence in different *Verticillium* strains was determined by PCR amplification on genomic DNA. As an endogenous control, a fragment of the *Verticillium ITS* region was amplified. The race 2 *V. dahliae* strain DVD S26 that lacks *Ave1* and water were used as PCR controls. The data shown are representative of two independent experiments.


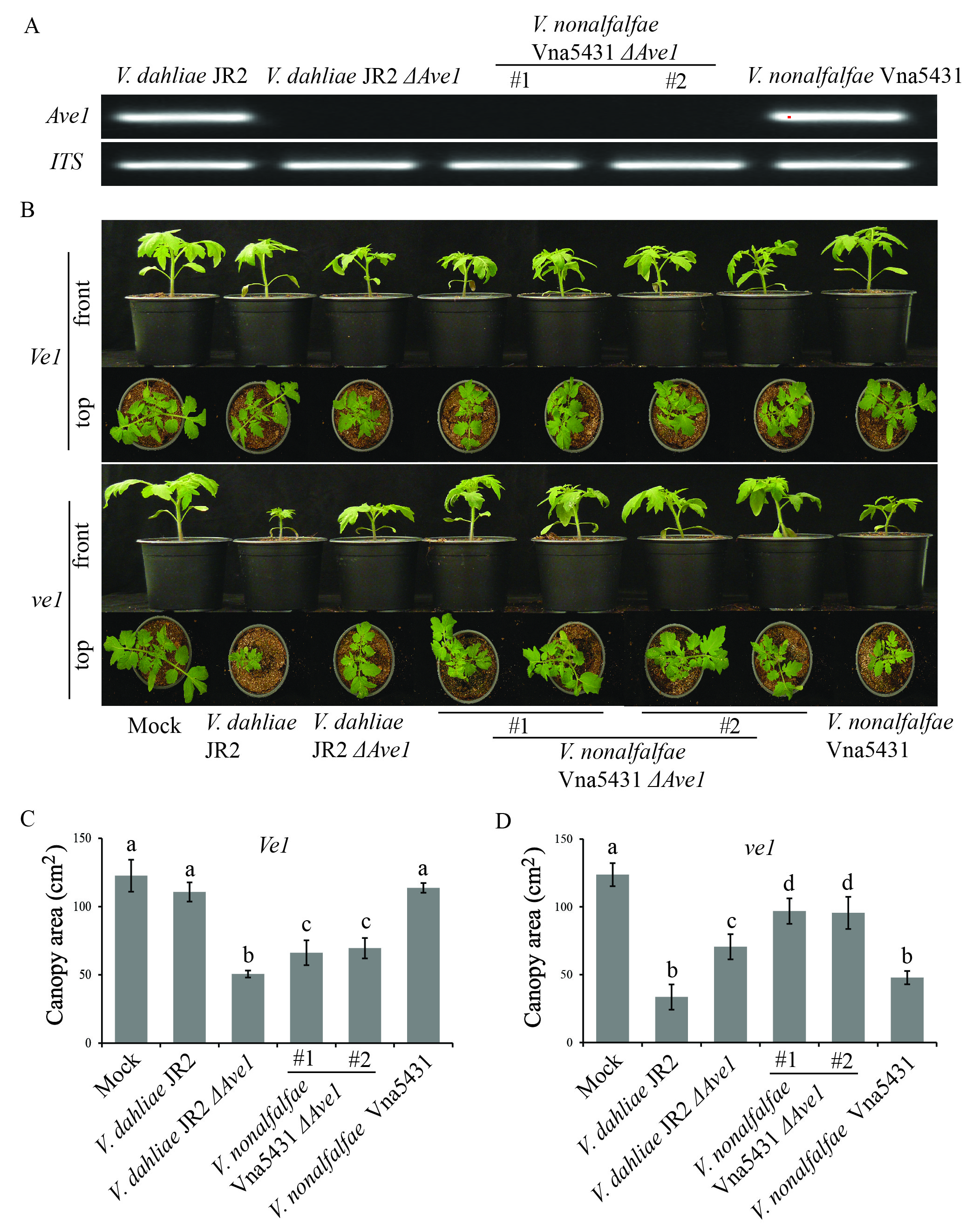


**Figure S3.** Analysis of *Ave1* deletion strains of *V. nonalfalfae* Vna5431. (A) Amplification of *Ave1* from genomic DNA in race 1 *V. dahliae* strain JR2, an *Ave1* deletion mutant of *V. dahliae* strain JR2 (Δ*Ave1*), race 1 *V. nonalfalfae* strain Vna5431 and two independent *Ave1* deletion strains (Δ*Ave1* #1 and #2). As an endogenous control, a fragment of the *Verticillium ITS* region was amplified. (B) Typical appearance of *Ve1* tomato plants (*Ve1*) and tomato plants lacking *Ve1* (*ve1*) upon mock-inoculation or inoculation with the various *Verticillium* strains at 14 days post inoculation (dpi). Average canopy area of eight *Ve1* (C) or *ve1* (D) tomato plants inoculated with the various *Verticillium* strains or mock-inoculation. Different letter labels indicate statistically significant differences (Student’s *t*-test; *P* < 0.05). The data shown are representative of two independent assays.


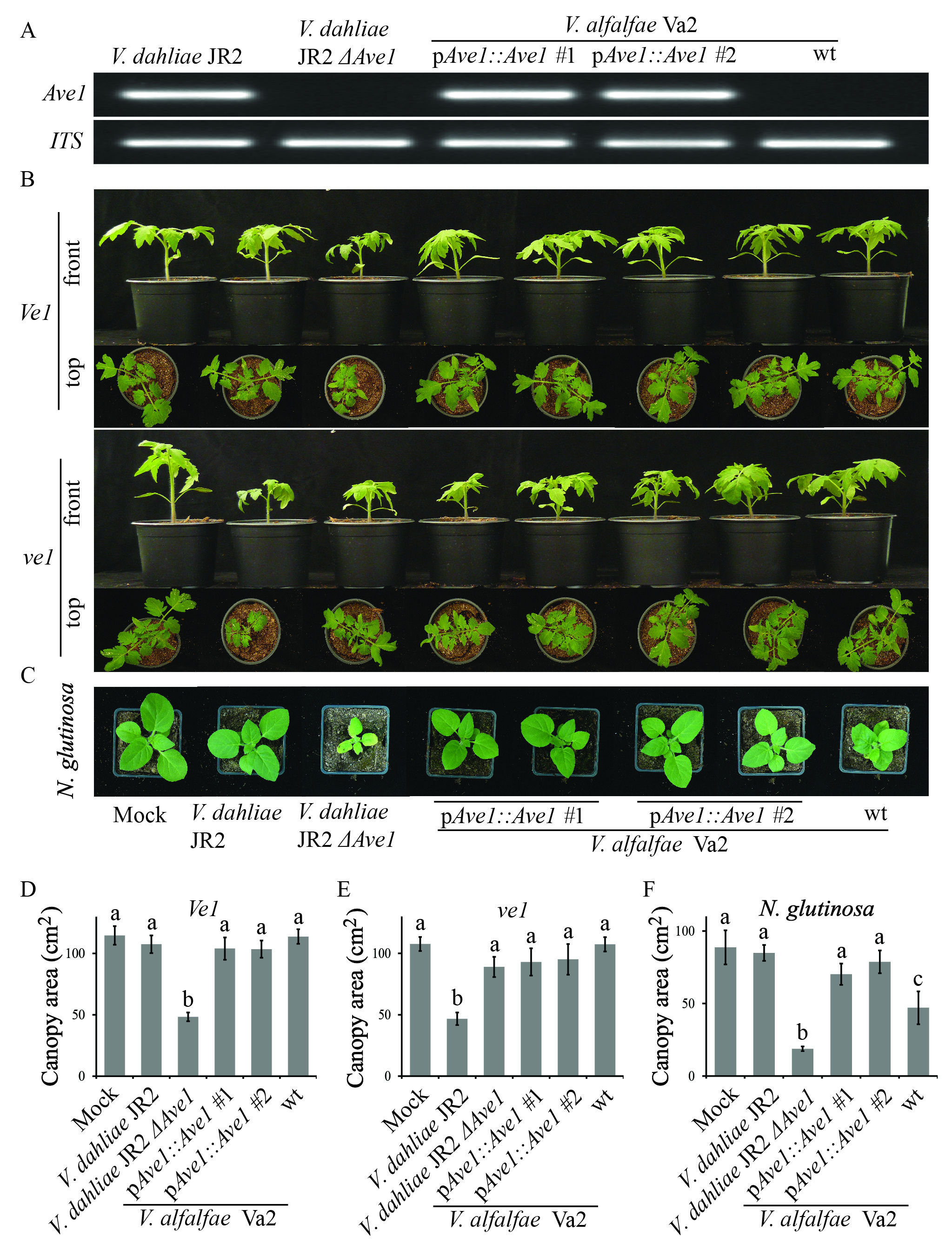


**Figure S4.** Analysis of ectopic expression *Ave1* strains in *V. alfalfae* Va2. (A) Amplification of *Ave1* from genomic DNA in race 1 *V. dahliae* strain JR2, an *Ave1* deletion mutant of *V. dahliae* strain JR2 (Δ*Ave1*), wild-type *V. alfalfae* strain Va2 (wt), and two independent *Ave1* expression strains (p*Ave1::Ave1* #1 and #2). As an endogenous control, a fragment of the *Verticillium ITS* region was amplified. (B) Typical appearance of *Ve1* tomato plants (*Ve1*) and tomato plants lacking *Ve1* (*ve1*) upon mock-inoculation or inoculation with the various *Verticillium* strains at 14 dpi. (C) Typical appearance of *Nicotiana glutinosa* plants upon mock-inoculation or inoculation with the various *Verticillium* strains at 14 dpi.Average canopy area of eight *Ve1* (D), *ve1* (E) tomato plants, or *N. glutinosa* plants (F) inoculated with the various *Verticillium* strainsor mock-inoculation. Different letter labels indicate statistically significant differences (Student’s *t*-test; *P* < 0.05). The data shown are representative of two independent assays.


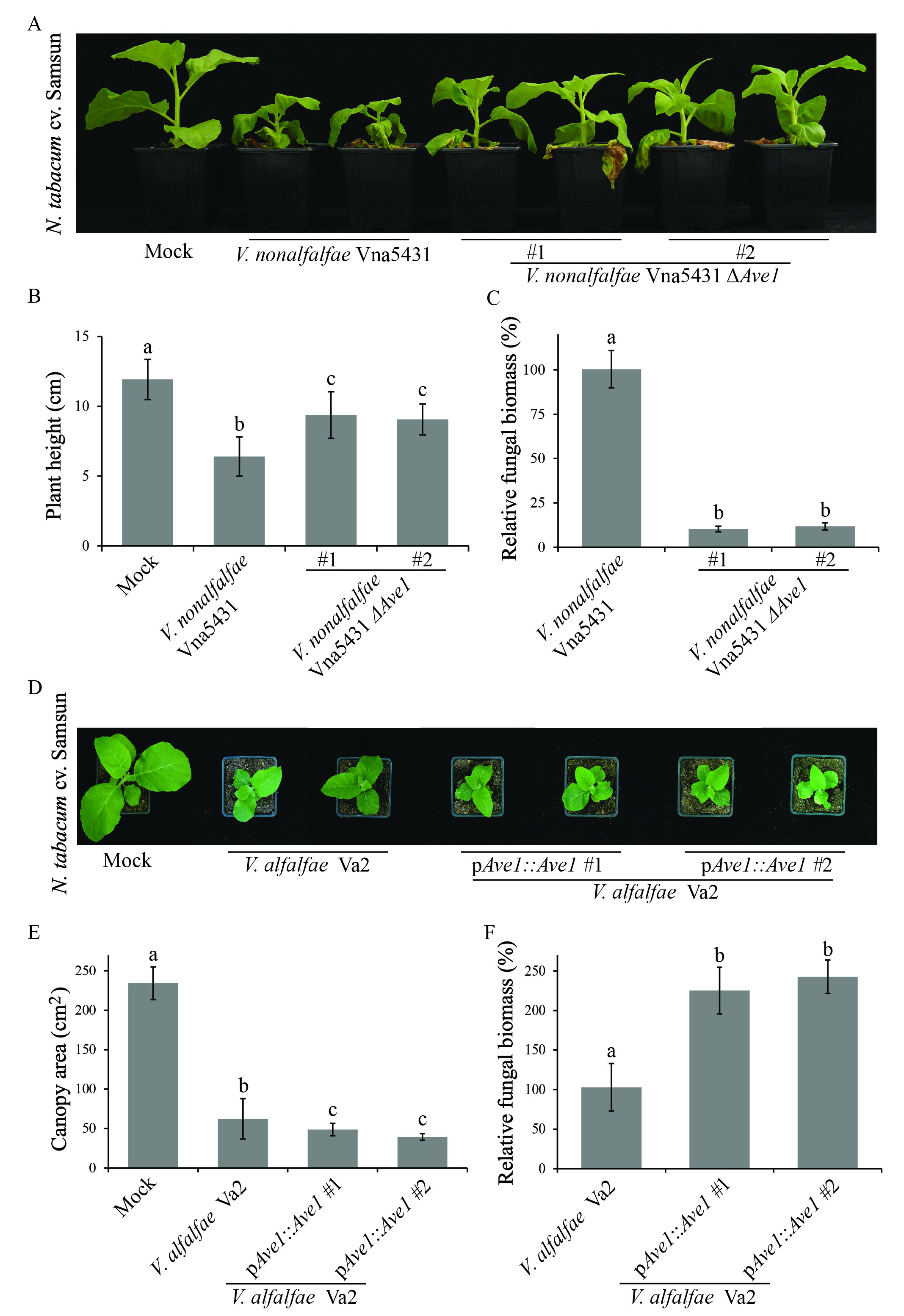


**Figure S5.** Ave1 acts as a virulence factor on tobacco cv. Samsun plants. (B) Typical appearance of tobacco cv. Samsun plants upon mock-inoculation, inoculation with *V. nonalfalfae* Vna5431, or two independent *Ave1* deletion strains (Δ*Ave1* #1 and #2) at 21 dpi. (B) Quantification of *Verticillium*-induced plant stunting at 21 dpi. Bars represent averages with standard deviation. (C) Fungal biomass as determined with real-time PCR at 21 dpi. Bars represent *Verticillium* *ITS* levels relative to tobacco *actin* levels (for equilibration) with standard deviation in a sample of three pooled plants. The fungal biomass in tobacco plants upon inoculation with the wild-type *V. nonalfalfae* strain Vna5431 is set to 100%. (D) Typical appearance of tobacco cv. Samsun plants upon mock inoculation, inoculation with *V. alfalfae* Va2, or two *Ave1*-experssing strains of *V. alfalfae* Va2 (p*Ave1::Ave1* #1 and #2) at 14 dpi. (E) Quantification of in the canopy area of tobacco cv. Samsun plants at 14 dpi. Bars represent averages with standard deviation. (C) Fungal biomass as determined with real-time PCR at 14 dpi. Bars represent *Verticillium* *ITS* levels relative to tobacco *actin* levels (for equilibration) with standard deviation in a sample of three pooled plants. The fungal biomass in tobacco cv. Samsun plants upon inoculation with the wild-type *V. alfalfae* strain Va2 is set to 100%. Different letter labels indicate statistically significant differences (Student’s *t*-test; *P* < 0.05). The data shown are representative of three independent assays.


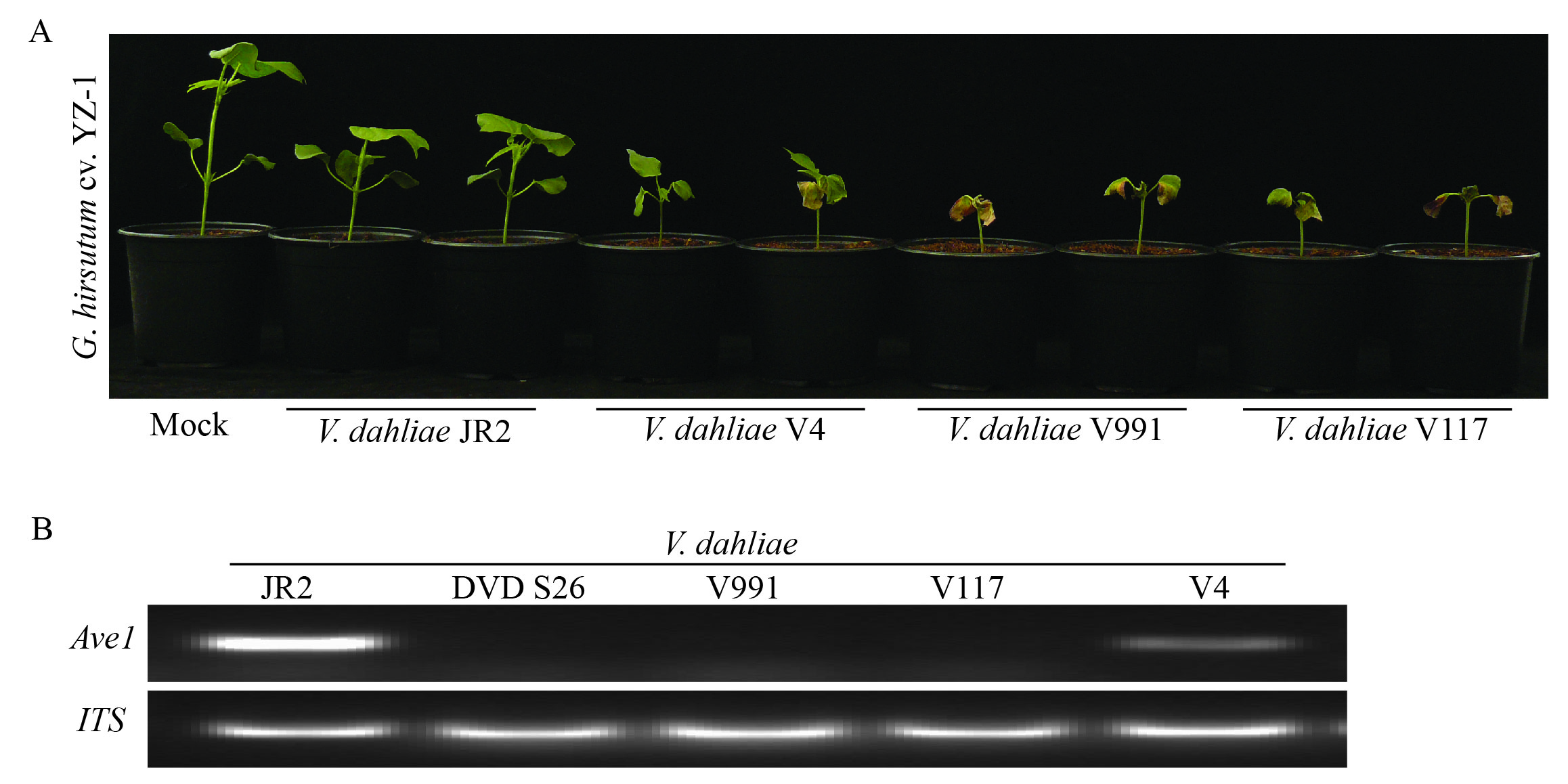


**Figure S6.** *V. dahliae* strains induce differential degrees of Verticillium wilt on cotton (*Gossypium hirsutum*) cv. YZ-1 plants. (A) Typical appearance of cotton cultivar YZ-1 plants upon mock-inoculation or inoculation with *V. dahliae* strains JR2, V4, V991 and V117 at 21 dpi. Inoculation experiments were performed with 10 plants for each *V. dahliae* strain and independently repeated twice. (B) Presence of the full-length *Ave1* coding DNA sequence was determined by PCR amplification on genomic DNA. As an endogenous control, a fragment of the *Verticillium ITS* region was amplified. The race 2 *V. dahliae* strain DVD S26 that lacks *Ave1* was used as a PCR control. The data shown are representative of two independent assays.


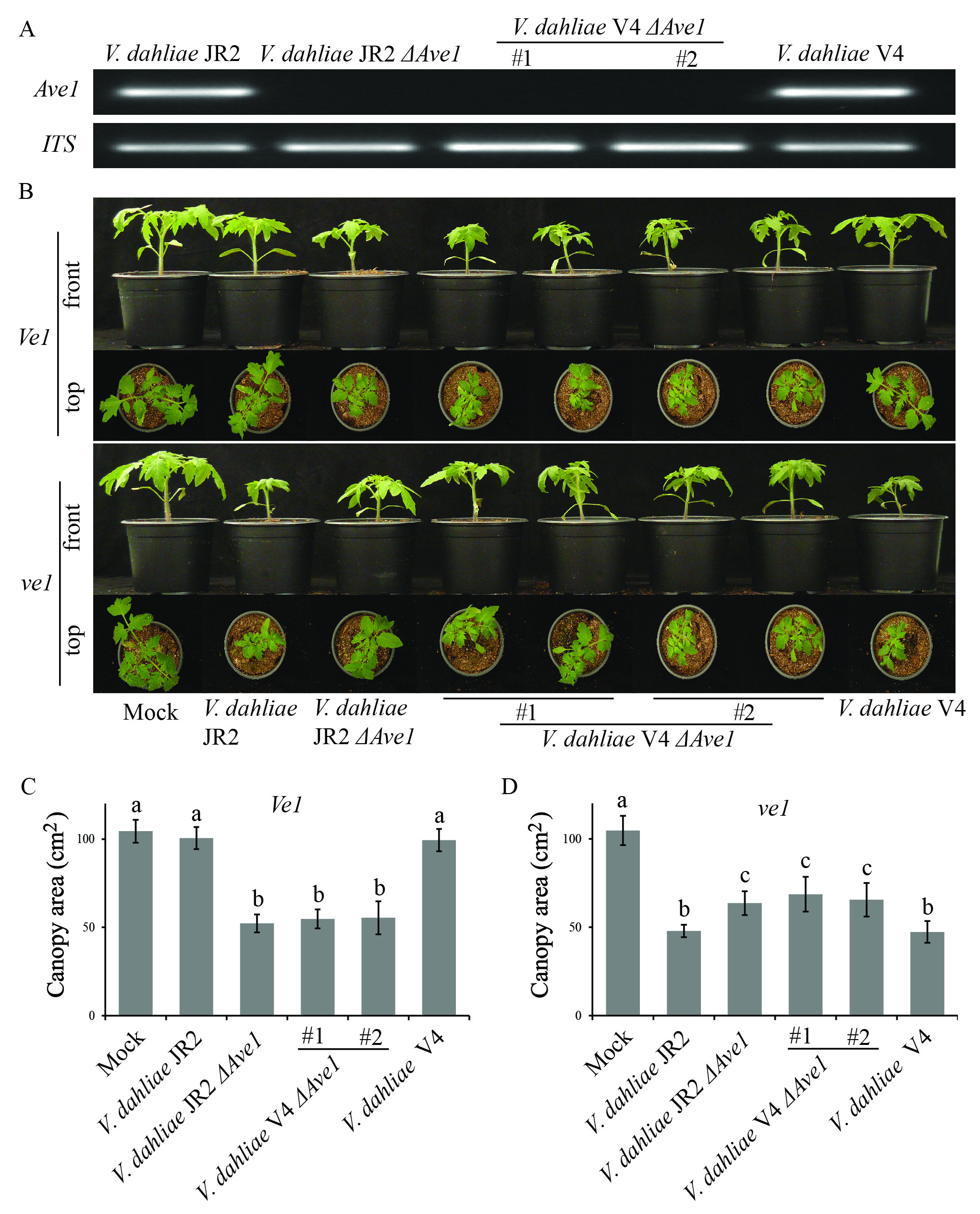


**Figure S7.** Analysis of *Ave1* deletion strains of *V. dahliae* V4. (A) Amplification of *Ave1* from genomic DNA in race 1 *V. dahliae* strain JR2, an *Ave1* deletion mutant of *V. dahliae* strain JR2 (Δ*Ave1*), race 1 *V. dahliae* strain V4 and two independent *Ave1* deletion strains (Δ*Ave1* #1 and #2). As an endogenous control, a fragment of the *Verticillium ITS* region was amplified. (B) Typical appearance of *Ve1* tomato plants (*Ve1*) and tomato plants lacking *Ve1* (*ve1*) upon mock-inoculation or inoculation with the various *V. dahliae* strains at 14 dpi. Average canopy area of eight *Ve1* (C) *ve1* (D) tomato plants inoculated with the various *V. dahliae* strains or mock-inoculation. Different letter labels indicate statistically significant differences (Student’s *t*-test; *P* < 0.05). The data shown are representative of two independent assays.


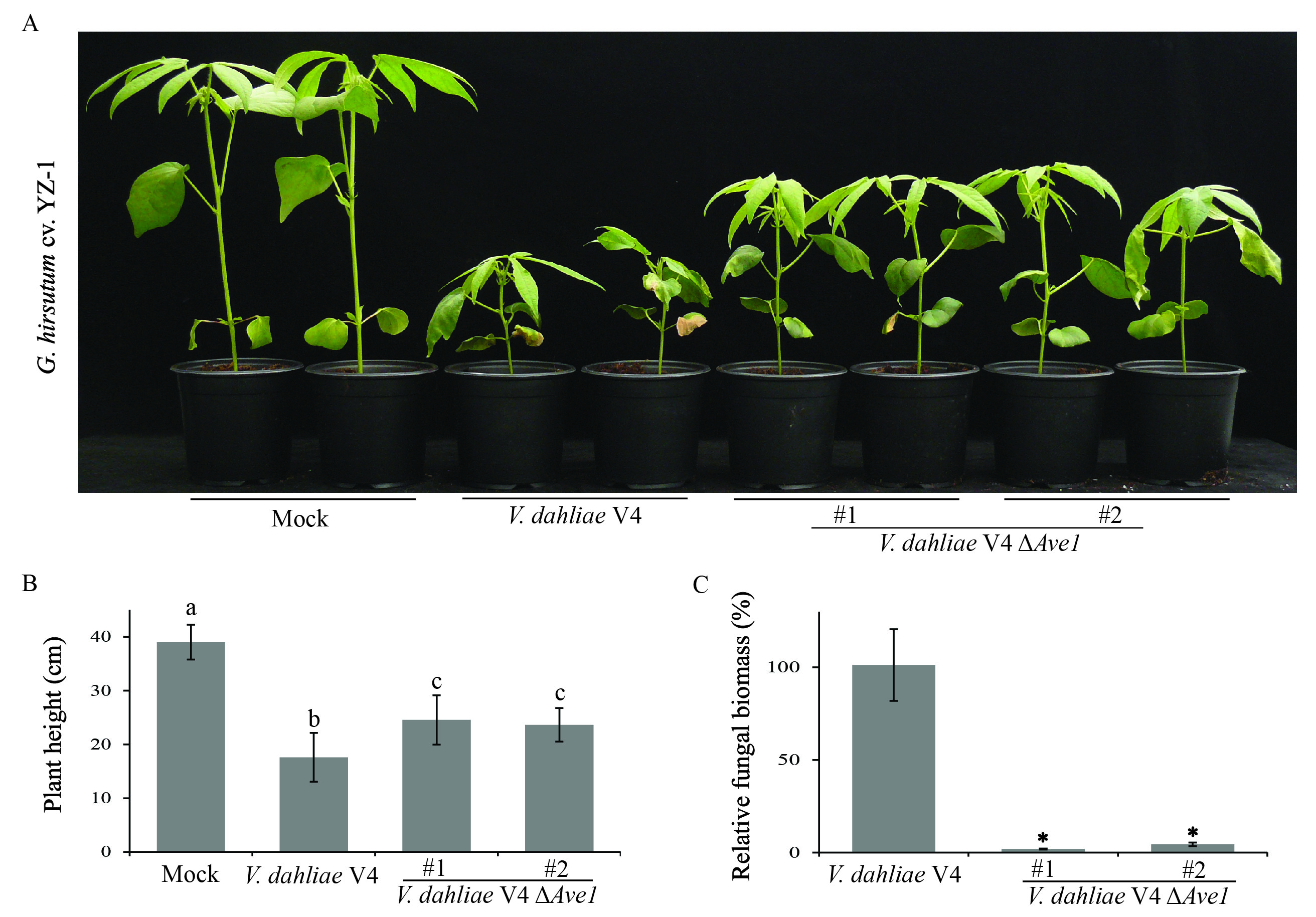


**Figure S8.** Ave1 acts as a virulence factor on cotton plants. (B) Typical appearance of cotton cultivar YZ-1 plants upon mock-inoculation or inoculation with *V. dahliae* V4 and two independent *Ave1* deletion strains (Δ*Ave1* #1 and #2) at 28 dpi. (B) Quantification of *Verticillium*-induced plant stunting at 28 dpi. Bars represent averages with standard deviation. Different letter labels indicate statistically significant differences (Student’s *t*-test; *P* < 0.05). (C) Fungal biomass as determined with real-time PCR at 28 dpi. Bars represent *Verticillium* *ITS* levels relative to cotton *ubiquitin* levels (for equilibration) with standard deviation in a sample of three pooled plants. The fungal biomass in cotton plants upon inoculation with the wild-type *V. dahliae* strain V4 is set to 100%. Asterisks indicate statistically significant differences when compared with cotton plants upon inoculation with the wild-type *V. dahliae* strain V4 (Student’s *t*-test; *P* < 0.05). The data shown are representative of three independent assays.

**Table S1. *Verticillium* strains used in this study**

| Strain | Description | Reference |
| --- | --- | --- |
| *V. dahliae* JR2 | Haploid, infecting tomato; *Ave1* presence | (de Jonge et al., 2012; Faino et al., 2015) |
| *V. dahliae* St14.01 | Haploid, infecting tomato; *Ave1* presence | (Fradin et al., 2009; de Jonge et al., 2012) |
| *V. dahliae* DVD S26 | Haploid, infecting tomato; *Ave1* absence | (de Jonge et al., 2012) |
| *V. nonalfalfae* Vna5431 | Haploid, isolated from tomato; *Ave1* presence;  Previously *V. albo-atrum* | (Fradin et al., 2009; Song et al., 2017); |
| *V. nonalfalfae* CBS385.91 | Haploid, isolated from tomato; *Ave1* presence;  Previously *V. albo-atrum* | (Fradin et al., 2009; Song et al., 2017) |
| *V. nonalfalfae* Vna1 | Haploid, original host is unkown; *Ave1* absence;  Previously *V. albo-atrum* | (Fradin et al., 2009; Song et al., 2017); |
| *V. alfalfae* Va2 | Haploid, original host is unkown; *Ave1* absence;  Previously *V. albo-atrum* | (Song et al., 2017); |
| *V. dahliae* V4 | Haploid, infecting cotton; *Ave1* presence | (López-Escudero et al., 2004; Song et al., 2017) |
| *V. dahliae* V117 | Haploid, infecting cotton; *Ave1* absence | (López-Escudero et al., 2004; Song et al., 2017) |
| *V. dahliae* V991 | Haploid, infecting cotton; *Ave1* absence | (Xu et al., 2014) |
| *V. dahliae* JR2 Δ*Ave1* | *Ave1* deletion mutant in *V. dahliae* JR2 | (de Jonge et al., 2012) |
| *V. nonalfalfae* Vna5431Δ*Ave1* #1 | *Ave1* deletion mutant in *V. nonalfalfae* Vna5431, colony 1 | This study |
| *V. nonalfalfae* Vna5431Δ*Ave1* #2 | *Ave1* deletion mutant in *V. nonalfalfae* Vna5431, colony 2 | This study |
| *V. alfalfae* Va2 p*Ave1::Ave1* #1 | *V. alfalfae* strain Va2 expressing *Ave1*, colony 1 | This study |
| *V. alfalfae* Va2 p*Ave1::Ave1* #2 | *V. alfalfae* strain Va2 expressing *Ave1*, colony 2 | This study |
| *V. dahliae* V4 Δ*Ave1* #1 | *Ave1* deletion mutant in *V. dahliae* V4, colony 1 | This study |
| *V. dahliae* V4 Δ*Ave1* #2 | *Ave1* deletion mutant in *V. dahliae* V4, colony 2 | This study |

**Table S2. Primers used in this study**

| Primer name | Oligonucleotide sequence (5’→3’) | Description^a^ |
| --- | --- | --- |
| Ve1-F(PCR) | CATATTGAAATTAGCGTCTTGTCGG | RT-PCR;  *Ve1* expression in tobacco |
| Ve1-R(PCR) | ACCGAGAAAAAGGAGGCAAAAC | RT-PCR;  *Ve1* expression in tobacco |
| Ave1-F | CACCATGAAGCTTTCTACGCTTGGAG | *Ave1*; PCR |
| Ave1-R | TTATATCTGTCTAAATTCGATGTTGAC | *Ave1*; PCR |
| ITS-F | AAAGTTTTAATGGTTCGCTAAGA | *Verticillium* ribosomal internal transcribed spacer region (ITS) |
| ITS-R | CTTGGTCATTTAGAGGAAGTAA | *Verticillium* ribosomal internal transcribed spacer region (ITS) |
| NtACT-F | CTATTCTCCGCTTTGGACTTGGCA | Tobacco *actin* |
| NtACT-R | AGGACCTCAGGACAACGGAAACG | Tobacco *actin* |
| GhUb-F | GAAGGCATTCCACCTGACCAAC | Cotton *ubiquitin* |
| GhUb-R | CAAAACTCCAAAATCATACCCAAAG | Cotton *ubiquitin* |
| Ve1-F(RT) | ATGGTTGCTGATGATTATGTGG | RT-PCR;  *Ve1* expression in cotton |
| Ve1-R(RT) | AATCAGGCAATGGTGTAGGTG | RT-PCR;  *Ve1* expression in cotton |

^a^The type of experiment for which the primers were used is indicated in brackets (RT-PCR: Reverse Transcription-PCR)

**REFERENCES**

de Jonge, R., van Esse, H.P., Maruthachalam, K., Bolton, M.D., Santhanam, P., Saber, M.K., Zhang, Z., Usami, T., Lievens, B., Subbarao, K.V. and Thomma, B.P.H.J. (2012) Tomato immune receptor Ve1 recognizes effector of multiple fungal pathogens uncovered by genome and RNA sequencing. *Proc. Natl Aca. Sci. USA*, **109**, 5110-5115.

Faino, L., Seidl, M.F., Datema, E., van den Berg, G.C., Janssen, A., Wittenberg, A.H. and Thomma, B.P.H.J. (2015) Single-Molecule Real-Time Sequencing Combined with Optical Mapping Yields Completely Finished Fungal Genome. *mBio*, **6**, e00936-00915.

Fradin, E.F., Zhang, Z., Ayala, J.C.J., Castroverde, C.D., Nazar, R.N., Robb, J., Liu, C.-M. and Thomma, B.P.H.J. (2009) Genetic dissection of Verticillium wilt resistance mediated by tomato Ve1. *Plant Physiol.* **150**, 320-332.

López-Escudero, F.J., del Río, C., Caballero, J.M. and Blanco-López, M.A. (2004) Evaluation of olive cultivars for resistance to *Verticillium dahliae*. *Eur. J. Plant Pathol.* **110**, 79-85.

Song, Y., Zhang, Z., Boshoven, J., Rovenich, H., Seidl, M., Jakse, J., Maruthachalam, K., Liu, C.-M., Subbarao, K., Javornik, B. and Thomma, B.P.H.J. (2017) Tomato immune receptor Ve1 recognizes surface-exposed co-localized N-and C-termini of *Verticillium dahliae* effector Ave1. *bioRxiv*, 103473.

Xu, L., Zhang, W., He, X., Liu, M., Zhang, K., Shaban, M., Sun, L., Zhu, J., Luo, Y., Yuan, D. Zhang, X., and Zhu, L. (2014) Functional characterization of cotton genes responsive to *Verticillium dahliae* through bioinformatics and reverse genetics strategies. *J. Exp. Bot.* **65**, 6679-6692.
